# The disjunct pattern of the Neotropical harvestman *Discocyrtus dilatatus* (Gonyleptidae) explained by climate-driven range shifts in the Quaternary: Paleodistributional and molecular evidence

**DOI:** 10.1371/journal.pone.0187983

**Published:** 2017-11-15

**Authors:** Julia Vergara, Luis E. Acosta, Raúl E. González-Ittig, Luis M. Vaschetto, Cristina N. Gardenal

**Affiliations:** 1 Instituto de Diversidad y Ecología Animal (IDEA), CONICET-Universidad Nacional de Córdoba, Argentina; 2 Cátedra de Diversidad Animal I, Facultad de Ciencias Exactas, Físicas y Naturales, U. N. C., Córdoba, Argentina; 3 Cátedra de Genética de Poblaciones y Evolución, Facultad de Ciencias Exactas, Físicas y Naturales, U. N. C., Córdoba, Argentina; Indiana University Bloomington, UNITED STATES

## Abstract

The disjunct distribution of the harvestman *Discocyrtus dilatatus* (Opiliones, Gonyleptidae) is used as a case study to test the hypothesis of a trans-Chaco Pleistocene paleobridge during range expansion stages. This would have temporarily connected humid regions (‘Mesopotamia’ in northeastern Argentina, and the ‘Yungas’ in the northwest, NWA) in the subtropical and temperate South American lowlands. The present study combines two independent approaches: paleodistributional reconstruction, using the Species Distribution Modeling method MaxEnt and projection onto Quaternary paleoclimates (6 kya, 21 kya, 130 kya), and phylogeographic analyses based on the *cytochrome oxidase subunit I* molecular marker. Models predict a maximal shrinkage during the warm Last Interglacial (130 kya), and the rise of the hypothesized paleobridge in the Last Glacial Maximum (21 kya), revealing that cold-dry stages (not warm-humid ones, as supposed) enabled the range expansion of this species. The disjunction was formed in the mid-Holocene (6 kya) and is intensified under current conditions. The median-joining network shows that NWA haplotypes are peripherally related to different Mesopotamian lineages; haplotypes from Santa Fe and Córdoba Provinces consistently occupy central positions in the network. According to the dated phylogeny, Mesopotamia-NWA expansion events would have occurred in the last glacial period, in many cases closely associated to the Last Glacial Maximum, with most divergence events occurring shortly thereafter. Only two (out of nine) NWA haplotypes are shared with Mesopotamian localities. A single, presumably relictual NWA haplotype was found to have diverged much earlier, suggesting an ancient expansion event not recoverable by the paleodistributional models. Different measures of sequence statistics, genetic diversity, population structure and history of demographic changes are provided. This research offers the first available evidence for the historical origin of NWA disjunct populations of a Mesopotamian harvestman.

## Introduction

Paleoenvironmental and distributional changes driven by climatic pulses during the Quaternary have long attracted the interest of biogeographers studying the biota of the Neotropics. This period was characterized by cyclic oscillations between glacial (cold) and interglacial (warm) stages that were often (though not always) correlated, respectively, to sub-xeric and humid conditions [1–3]. As numerous palynological studies support, climatic fluctuations induced vegetation shifts in tropical and subtropical South America causing forests to expand during humid periods and regress during dry stages, the latter resulting in forests being replaced by open, xeric or more seasonal formations [4–9]. In some areas, the expansion events had the recurrent effect of enabling the connectivity between forested regions that were otherwise separated by non-forested (e.g. grassland) or xeric areas. Therefore, dispersal or interchange of forest-dwelling species was transitory, eventually generating disjunct patterns such as those observed between the Brazilian Amazonian and Atlantic forests, which were connected by forest corridors during expansion phases across the current seasonally dry Cerrado, a savanna-type biome ([10–12] and references therein).

**Fig 1:**
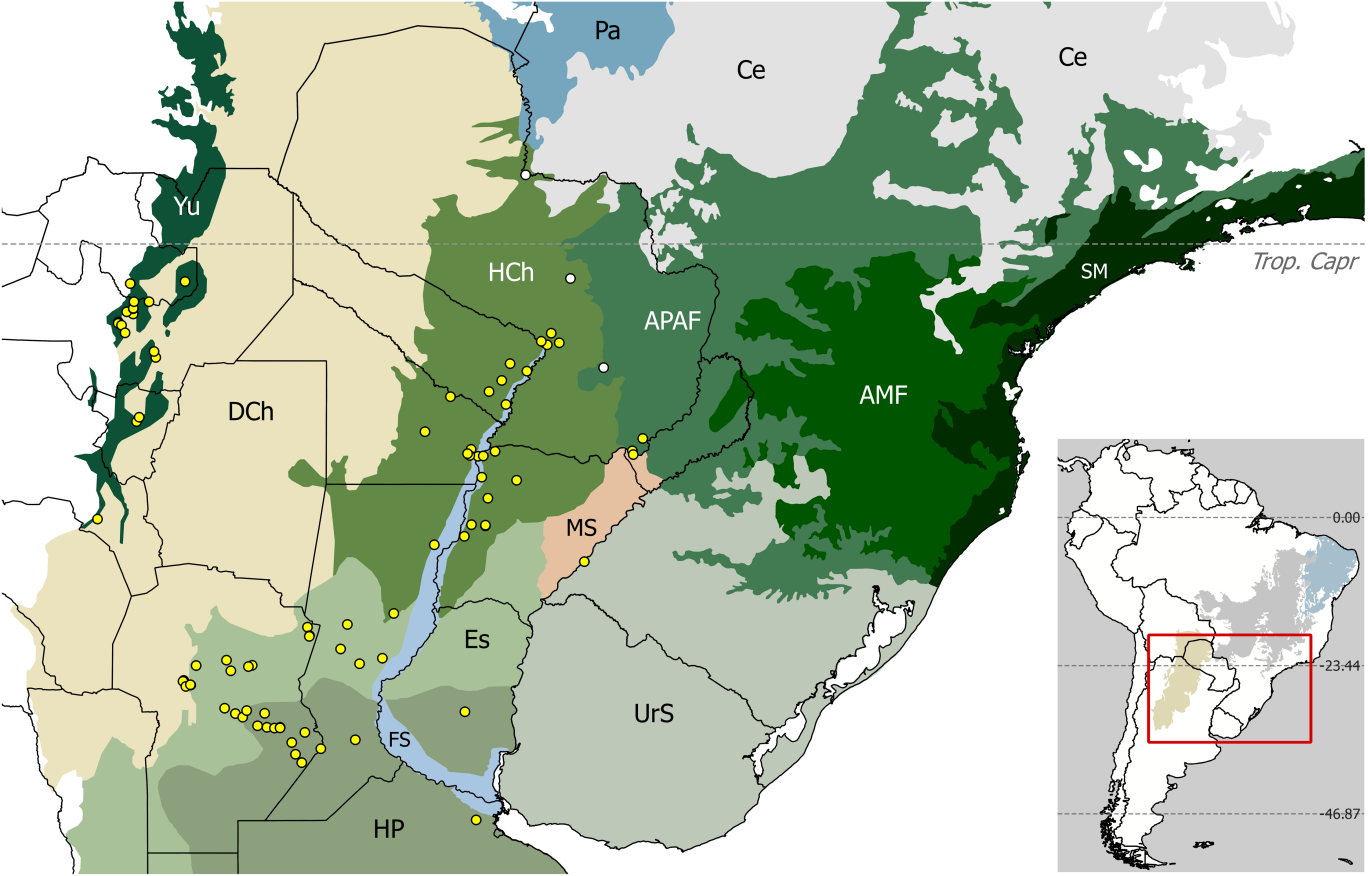
Presence records of *Discocyrtus dilatatus* plotted over relevant ecoregions in subtropical and temperate South America. Yellow dots: records used to calibrate the models; white dots (in Paraguay): three pre-1950 records excluded in this research. Dashed line: Tropic of Capricorn (Trop. Capr, 23°26’14”S). Nomenclature of ecoregions [14]: HCh: Humid Chaco, DCh: Dry Chaco, Yu: Southern Andean Yungas, FS: Paraná flooded savanna, Es: Espinal, HP: Humid Pampas, MS: Southern Cone Mesopotamian savanna, UrS: Uruguayan savanna, APAF: Alto Paraná Atlantic forests, AMF: Araucaria moist forests, SM: Serra do Mar coastal forests, Pa: Pantanal, Ce: Cerrado. Inset: sector represented in South America, displaying the ecoregions that form the ‘dry diagonal’ of open vegetation (Dry Chaco-yellow, Cerrado-grey, Caatinga-blue). Maps were designed using free spatial data available at http://www.diva-gis.org/Data, and https://www.worldwildlife.org/publications/terrestrial-ecoregions-of-the-world.

Further south, an analogous pattern was recognized at the subtropical–temperate portion of the so-called ‘dry diagonal’ of open vegetation (Fig 1) [10–13]. There, the sub-xeric Dry Chaco ecoregion [14] constitutes an extensive barrier between humid ecoregions: the Yungas (sub-Andean rain forests in north-western Argentina, henceforth NWA) on the west side, and the Alto Paraná Atlantic Forests (or Paranense forests) plus the Humid Chaco on the east (Fig 1) [15–17]. Several plant species, with separated populations in both humid areas, illustrate this ‘trans-Chaco disjunction’ well (e.g., [18–21]). To account for disjunct avian distributions in this area, a forested ‘paleobridge’ along the Pilcomayo and BermejoRivers basins was hypothesized [15, 16]. This corridor, herein referred to as ‘Nores´ paleobridge’, would have connected the Paranense and the Yungas forests several times during humid–warm interglacials in the Pleistocene. In this way, it might have allowed the range expansion of forest-dwelling birds, followed by the separation and isolation of populations as humid forests regressed during dry–cold glacial stages [15, 16].

Nores’ hypothesis seemed pertinent to explain the findings in NWA of disjunct populations of wide-ranged Mesopotamian and Paranense harvestmen: the gonyleptids *Discocyrtus dilatatus* Sørensen, 1884 (Fig 1), *D*. *prospicuus* (Holmberg, 1876) and *Geraeocormobius sylvarum* Holmberg, 1887, and the cosmetid *Gryne orensis* (Sørensen, 1879) [22–27]. As widely recognized, harvestmen are heavily dependent on humid environments [23, 28–30]; consequently, the 450 to 650 km gap of inhospitable Dry Chaco represents an insurmountable barrier to active dispersal for such low-vagility organisms, at least under current conditions. The ‘Mesopotamian *sensu stricto*’ opiliogeographical area [23] extends over the vast alluvial plains around the fluvial ‘hub’ formed by the Paraguay River and the middle and lower Paraná River, a formidable dispersal route that connects humid tropical with temperate regions [31]. The core of the Mesopotamian *s*.*s*. area coincides with the Humid Chaco ecoregion (Fig 1)[14, 32], there by extending on the eastern margins of the Argentinean provinces of Formosa, Chaco and northern Santa Fe, up to the environmental limits imposed by the Dry Chaco. Mesopotamian harvestmen also spread further south over the Espinal scrubland and the borders of the Pampean steppe, from which a few species stretch in a prominent westward expansion to reach the base of the Córdoba Sierras [23]. The northeastern-most Argentinean province (i.e., Misiones) is covered by Paranense rainforests and constitutes a separate opiliogeographical unit. The fact that the Mesopotamia-NWA (and Paranense-NWA) disjunct pattern was observed in more than one entity was deemed to strengthen evidence for Nores’ paleobridge [26]. However, at least for the Paranense-NWA disjunction of *G*. *sylvarum* neither paleodistributional nor preliminary molecular analyses supported this historical explanation, rather suggesting a recent anthropic introduction [24, 33, 34]. In any case, the existence of a hypothetical trans-Chaco corridor was never formally tested for a Mesopotamian harvestman. Given its known range (Fig 1), *Discocyrtus dilatatus*, a kind of ‘flagship species’ for the disjunction case [23, 27], appears particularly well-suited to test the paleobridge hypothesis. In this paper, this question is tackled by means of two independent, complementary approaches, as detailed below.

The first approach consists of the Species Distribution Modeling (SDM) based on climatic predictors, a robust way to address the question of whether a ‘paleoconnection’ could be reconstructed for *D*. *dilatatus*. SDM is a widely used methodology which, among other strengths, is designed to cope with the incompleteness or paucity of record data [35–37], as is the case for most Neotropical harvestmen. Some Mesopotamian species have been the subject of spatial analysis through SDM, but those analyses were based on current climate alone (*Gryne orensis* [26], *Discocyrtus testudineus* (Holmberg, 1876) [38], *D*. *dilatatus* [27]). Provided that paleoclimatic information is available, climate-based SDMs are feasible to be projected onto past chronologies to estimate paleodistributions [12; 39–42]. Thus, one major goal of this paper is to hindcast distributional shifts of *D*. *dilatatus* for three Upper Quaternary stages, as representative periods of the changing climatic conditions: Last Interglacial (LIG, ~130 kya = -130k; warm), Last Glacial Maximum (LGM, ~21 kya = -21k; cold), and Holocene climatic optimum (HCO, ~6 kya = -6k; mild). Since availability of humid environments is a relevant constraint for Mesopotamian harvestmen [23, 28], *D*. *dilatatus* was primarily expected to behave in accordance to Nores’ postulates [15, 16], i.e., expanding during the humid-warm interglacials and shrinking in the dry-cold glacial stages. The aim was then to examine whether paleodistributions are likely to have followed such a straightforward prediction, as to reconstruct the past trans-Chaco connectivity.

If the presence of a Mesopotamian species in NWA is the result of a historical event (rather than, for example, anthropic introduction or a recent natural dispersal), traces of that are expected to be found within its population genetic structure. This aspect is hereby tested through the phylogeography approach, which provides a sound analytical framework to study the processes and principles that govern the geographical distribution of intraspecific genealogical lineages [43, 44]. Phylogeography provides powerful tools to investigate genetic population changes at the Quaternary time scale [45], and thus is useful to reciprocally contrast results of the paleoclimate models [42, 46, 47]. The vast majority of species in nature exhibit some degree of genetic structuring associated with geography, which can be explained by paleoenvironmental causes (such as tectonics, volcanic activity or paleoclimatic changes) or, more simply, by high migration rates or relatively recent isolation [43, 44]. The phylogeographic analysis may help to uncover evolutionary events at the species level, such as habitat fragmentation, range expansion, migration, vicariance or lineage extinction. Several contributions have already demonstrated the usefulness of this methodological approach to tackle biogeographic questions in harvestmen [48–52]. In this paper, an integrated SDM / phylogeographic approach is proposed to evaluate the historical scenarios invoked to explain the disjunct pattern of *D*. *dilatatus*. This research is conceived as a case study, to contribute to understanding the processes that may have shaped some major biogeographical patterns in the lowlands of sub-tropical and temperate South America.

## Material and methods

### Paleodistribution models

#### Records of *Discocyrtus dilatatus*

The original dataset, detailed in [27], included 85 georeferenced unique localities (Fig 1). For this analysis, the reliability of pre-1950 records was revisited, retaining only those validated by recent collections, at least from surrounding localities; this resulted in three points from Paraguay being excluded (Fig 1). After merging duplicate records shared by the same grid-cell, 77 presence points remained effective records for modeling; their coordinates are available in S1 File (.csv format).

#### Climate layers

Climatic grids at a resolution of 2.5 arc-min were employed, delimited between 74.6667°W / 35.2083°W, and 14.1250°S / 41.8333°S. Slices for current climate were obtained from the WorldClim 1.4 free climate database (http://www.worldclim.org/), which comprises values for 19 bioclimatic (bc) variables, averaging the 1950–2000 period [53]. Spatial correlation of variables (Pearson>0.80) was prevented prior to the modeling process, to reduce overparameterization [17, 54]; the pairwise correlation was calculated for every combination of raster files, separately for temperature and precipitation, using the software ENMTools 1.3 [55]. Since the ecophysiological meaning of most bc variables is unknown [30], given two or more correlated variables, the one ranking higher for the ‘sum score’ (percent contribution + permutation importance), in a preliminary MaxEnt run with all 19 bc, was retained for modeling [26, 38]. As a result, 10 bioclimatic predictors were used: (bc2) mean monthly temperature range; (bc3) isothermality; (bc4) temperature seasonality; (bc5) maximum temperature of warmest month; (bc9) mean temperature of driest quarter; (bc13) precipitation of wettest month; (bc14) precipitation of driest month; (bc15) precipitation seasonality; (bc18) precipitation of warmest quarter; (bc19) precipitation of coldest quarter.

The bioclimatic niche model, calibrated for current conditions, was projected on paleoclimate layers representing the mid-Holocene or Holocene climatic optimum (HCO, -6k), the Last Glacial Maximum (LGM, -21k) and the Last inter-glacial (LIG, -130k). As a part of the Last Glacial period (also known as Würm glaciation), the LGM was a stage of global cooling and formation of extensive glaciers. In contrast, mid-Holocene and LIG represent periods of climate amelioration; LIG, also referred to as the ‘Eemian’ period or Marine Isotope Stage 5e, was probably warmer than today [56, 57]. Paleoclimatic layers were downloaded from the WorldClim website (http://www.worldclim.org/), which include downscaled climate data from different Global Climate Models (GCMs), based on original data made available by CMIP5 (Coupled Model Intercomparison Project Phase 5; http://cmip-pcmdi.llnl.gov/cmip5/) [58]; data were calibrated using WorldClim 1.4 as baseline 'current' climate. The following GCM simulations were used, for both -6k and -21k: CCSM4, Community Climate System Model (‘CCSM’ from now on), National Center for Atmospheric Research; MIROC-ESM, Model for Interdisciplinary Research on Climate (‘MIROC’), Japan Agency for Marine-Earth Science and Technology, Atmosphere and Ocean Research Institute, The University of Tokyo, and National Institute for Environmental Studies; and MPI-ESM-P (‘MPI’), Max-Planck-Institut für Meteorologie. Paleoclimatic surfaces for LIG, obtained from WorldClim too, are based on [59].

#### Calibration of models

Models were built using the maximum-entropy algorithm MaxEnt (thoroughly described in [60, 61]). Version 3.3.3k of the software MaxEnt (http://www.cs.princeton.edu/~schapire/maxent) was employed. Besides ranking among the best performing methods [61], MaxEnt has been extensively used to project niche models onto paleoclimatic conditions (e.g., [10, 40, 41, 62]). To prevent over-complexity and/or overfitting of the final output, the optimal settings to calibrate the models were assessed in previous tuning experiments with a combination of two main adjustable MaxEnt parameters: feature class (linear, L, quadratic, Q, product, P, threshold, T, or hinge, H) and regularization multiplier [63–66]. Following the procedure outlined by [54], particularly useful for small sample sizes, the ‘best model’ parameters were selected by comparing 85 possible outputs, derived from 17 feature combinations and regularization multiplier varied by a step of 1, between the values of 1–5 (S1 Table). Since L features are special cases of H, any L+H combination was disregarded to avoid redundancy [63]. The use of regularization values closer to the default (= 1) was aimed to explore the possibility of relatively small changes affecting model output [66]. The resulting models, of varying complexity, were compared using the ‘sample size corrected Akaike information criteria’ (AICc) [64], implemented in ENMTools 1.3. The lowest AICc value (‘best model’) corresponded to QT-1 (quadratic + threshold features, and the default regularization multiplier); considering that [54], on almost the same record set, found QT-2 to be their best model, intermediate values (1.25, 1.50, 1.75) were further explored, resulting in the best model QT-1.25 (S1 Table), adopted as the final settings. Logistic output was selected. ‘Maximum iterations’ were raised to 2500. Prediction maps represent the mean values of a run comprised of 30 replicates for each chronology/GCM (current, -6k-CCSM, -6k-MIROC, -6k-MPI, LGM-CCSM, LGM-MIROC, LGM-MPI and LIG), trained with 90% of the points randomly selected (‘random seed’ active) and applying the ‘subsample’ replication type. To determine the boundary between ‘suitable’ versus ‘not suitable’ grid-cells, also to obtain binary (‘thresholded’) predictions, the ‘10 percentile training presence logistic threshold’ was adopted (recommended elsewhere [54], also resulting in more conservative projections). The employ of more than one climatic model for -6k and LGM is considered useful to reduce the uncertainty derived from variations among different GCMs [67]; output maps are displayed either individually or after applying some kind of consensus between CCSM-MIROC-MPI results (grids were averaged for logistic predictions, stacked for binary). Accuracy of the models was measured with the AUC (Area Under Curve) statistics, provided in MaxEnt by default; values were considered a ‘good’ model performance if greater than 0.8, or ‘high’ if greater than 0.9 [68].

#### Range shifts and stable areas

Changes in the extent and position of the species range in the studied chronologies were inspected through the overlay of binary models. Grid-cells shared by different chronologies were considered to represent ‘stable’ areas, i.e., deemed to have kept their suitability across the corresponding time slices [10, 62].

### Phylogeographic analyses

#### Samples

The molecular analyses were made on 181 specimens from 44 localities, covering most of the known range of *D*. *dilatatus* [22, 27]. The focal species is abundant at the localities surveyed in this study and is not endangered. Collection of specimens did not involve any protected area, and in most sites no specific permissions were required. Samples from Misiones Province were obtained under a collecting permit issued by E.R. Krauczuk (Ministerio de Ecología, R. N. R. y T.) in agreement to Res. N° 509/07. Specimens were deposited in the “Colección Aracnológica, Cátedra de Diversidad Animal I, Facultad de Ciencias Exactas, Físicas y Naturales, Universidad Nacional de Córdoba, Córdoba, Argentina, freezer collection” (CDA-F), preserved in 96% ethanol and maintained at -20°C until DNA extraction. CDA is a public permanent repository hosted at Córdoba National University, open to scientific research (Curator: L.E. Acosta, luis.acosta@unc.edu.ar). A list of the studied localities, with information of each sequenced specimen, including their GenBank accession number and CDA-F identifiers, is given in Table 1.

**Table 1:** Localities of *Discocyrtus dilatatus* studied for COI, with geographical coordinates, haplotypes detected, collection identifiers (CDA-F id#) and GenBank accession numbers.

Province	Locality	Longitude (W)	Latitude (S)	§	Haplotype	CDA-F id#	GenBank #
**Misiones**	Posadas	-55.90517	-27.36300	**M**	Dd.5	Dd-PO-A	MF168411
						Dd-PO-B	MF168412
						Dd-PO-C	MF168413
	Posadas (University Campus)	-55.88852	-27.43372	**M**	Dd.3	Dd-Po2-B	MF168415
					Dd.5	Dd-Po2-A	MF168414
					Dd.19 (U)	Dd-Po2-C	MF168416
**Corrientes**	Laguna Soto	-58.73500	-27.46000	**Cr**	Dd.2	Dd-LS-E	MF168421
						Dd-LS-F	MF168422
					Dd.3	Dd-LS-A	MF168417
						Dd-LS-D	MF168420
					Dd.4 (U)	Dd-LS-B	MF168418
						Dd-LS-C	MF168419
	Comuna 2 de abril	-58.69408	-28.77448	**Cr**	Dd.34 (U)	Dd-2A-A	MF168423
						Dd-2A-C	MF168425
					Dd.35 (U)	Dd-2A-B	MF168424
						Dd-2A-D	MF168426
	Saladas	-58.64737	-28.26248	**Cr**	Dd.1	Dd-Sa-B	MF168428
						Dd-Sa-C	MF168429
						Dd-Sa-D	MF168430
					Dd.36 (U)	Dd-Sa-A	MF168427
	Bridge over highroad 123	-58.95865	-28.76503	**Cr**	Dd.1	Dd-123-A	MF168431
						Dd-123-B	MF168432
						Dd-123-C	MF168433
						Dd-123-D	MF168434
						Dd-123-E	MF168435
	Río Empedrado, by RN12 bridge	-58.76385	-27.86338	**Cr**	Dd.3	Dd-RE-A	MF168436
						Dd-RE-B	MF168437
						Dd-RE-D	MF168438
**Formosa**	Colonia Dalmacia	-57.90703	-25.85018	**F**	Dd.20 (U)	Dd-CD-A	MF168439
					Dd.21 (U)	Dd-CD-B	MF168440
	Monte Lindo Grande	-58.22598	-25.70803	**F**	Dd.25 (U)	Dd-MLG-A	MF168441
						Dd-MLG-B	MF168442
						Dd-MLG-D	MF168444
						Dd-MLG-E	MF168445
						Dd-MLG-F	MF168446
					Dd.26 (U)	Dd-MLG-C	MF168443
	San Francisco de Laishi	-58.62548	-26.23843	**F**	Dd.24 (U)	Dd-La-A	MF168447
	Herradura (La Florencia camping site)	-58.30385	-26.48253	**F**	Dd.23 (U)	Dd-H-B	MF168448
						Dd-H-C	MF168449
						Dd-H-D	MF168450
						Dd-H-E	MF168451
						Dd-H-F	MF168452
	El Colorado	-59.35818	-26.33562	**F**	Dd.22	Dd-EC-A	MF168453
						Dd-EC-B	MF168454
						Dd-EC-C	MF168455
						Dd-EC-E	MF168456
						Dd-EC-F	MF168457
**Chaco**	Colonia Benítez, bridge	-58.96612	-27.33522	**Ch**	Dd.14	Dd-CB-D	MF168461
					Dd.27 (U)	Dd-CB-A	MF168458
					Dd.28 (U)	Dd-CB-B	MF168459
					Dd.29 (U)	Dd-CB-C	MF168460
						Dd-CB-E	MF168462
					Dd.37 (U)	Dd-CB-F	MF168463
**Santa Fe**	Reconquista	-59.66433	-29.14667	**C-SF**	Dd.13	Dd-R-A	MF168467
						Dd-R-B	MF168468
						Dd-R-C	MF168469
						Dd-R-D	MF168470
						Dd-R-E	MF168471
	Constanza	-61.31530	-30.65925	**C-SF**	Dd.13	Dd-Co-A	MF168472
						Dd-Co-B	MF168473
						Dd-Co-C	MF168474
						Dd-Co-D	MF168475
						Dd-Co-F	MF168476
						Dd-Co-G	MF168477
	Lehmann	-61.44297	-31.12453	**C-SF**	Dd.6	Dd-Le-D	MF168481
					Dd.13	Dd-Le-E	MF168482
					Dd.14	Dd-Le-A	MF168478
						Dd-Le-B	MF168479
						Dd-Le-C	MF168480
	Carcarañá	-61.16417	-32.84917	**C-SF**	Dd.1	Dd-Ca-A	MF168483
					Dd.12	Dd-Ca-B	MF168484
**Córdoba**	Morteros	-62.07200	-30.71000	**C-SF**	Dd.16	Dd-Mor-A	MF168485
						Dd-Mor-B	MF168486
	Brinkmann (backyard)	-62.03272	-30.87203	**C-SF**	Dd.14	Dd-Br-A	MF168487
						Dd-Br-B	MF168488
						Dd-Br-C	MF168489
						Dd-Br-D	MF168490
	Brinkman (plant nursery)	-62.03742	-30.88530	**C-SF**	Dd.14	Dd-Br-E	MF168491
						Dd-Br-F	MF168492
						Dd-Br-G	MF168493
					Dd.16	Dd-Br-H	MF168494
	Río Primero	-63.44267	-31.33733	**C-SF**	Dd.1	Dd-RP-A	MF168495
						Dd-RP-B	MF168496
					Dd.2	Dd-RP-C	MF168497
						Dd-RP-D	MF168498
						Dd-RP-F	MF168499
						Dd-RP-H	MF168500
	Villa del Rosario	-63.52667	-31.53333	**C-SF**	Dd.2	Dd-VR-B	MF168502
					Dd.6	Dd-VR-A	MF168501
						Dd-VR-C	MF168503
						Dd-VR-D	MF168504
						Dd-VR-E	MF168505
						Dd-VR-F	MF168506
	Villa Los Aromos	-64.43533	-31.73867	**C-SF**	Dd.3	Dd-LA-A	MF168507
	Los Molinos	-64.38117	-31.83617	**C-SF**	Dd.11	Dd-LM-A	MF168508
						Dd-LM-B	MF168509
					Dd.13	Dd-LM-C	MF168510
						Dd-LM-E	MF168511
						Dd-LM-F	MF168512
	Despeñaderos 1 (riverside)	-64.28800	-31.80900	**C-SF**	Dd.3	Dd-De1-A	MF168513
						Dd-De1-B	MF168514
	Despeñaderos 2 (near bridge)	-64.29500	-31.80400	**C-SF**	Dd.3	Dd-De2-A	MF168515
						Dd-De2-B	MF168516
						Dd-De2-C	MF168517
						Dd-De2-D	MF168518
						Dd-De2-F	MF168519
	Pampayasta Sur	-63.64483	-32.24917	**C-SF**	Dd.6	Dd-PY-A	MF168520
						Dd-PY-B	MF168521
						Dd-PY-C	MF168522
						Dd-PY-E	MF168524
						Dd-PY-F	MF168525
					Dd.12	Dd-PY-D	MF168523
	Estancia Yucat	-63.44083	-32.35138	**C-SF**	Dd.6	Dd-Yu-A	MF168526
						Dd-Yu-B	MF168527
	Villa María, bridge highroad to Río IV	-63.30183	-32.41967	**C-SF**	Dd.2	Dd-VM-A	MF168528
					Dd.7	Dd-VM-C	MF168530
					Dd.13	Dd-VM-B	MF168529
					Dd.14	Dd-VM-D	MF168531
	Ballesteros Sur	-63.01933	-32.58300	**C-SF**	Dd.11	Dd-BS-A	MF168532
						Dd-BS-B	MF168533
						Dd-BS-D	MF168535
					Dd.12	Dd-BS-C	MF168534
						Dd-BS-E	MF168536
	Morrison	-62.84110	-32.61200	**C-SF**	Dd.3	Dd-MO-C	MF168539
					Dd.7	Dd-MO-A	MF168537
					Dd.8 (U)	Dd-MO-B	MF168538
					Dd.9 (U)	Dd-MO-D	MF168540
	Marcos Juárez	-62.12117	-32.70833	**C-SF**	Dd.13	Dd-MJ-A	MF168541
						Dd-MJ-B	MF168542
						Dd-MJ-C	MF168543
	Monte Buey	-62.37033	-32.90060	**C-SF**	Dd.3	Dd-MB-A	MF168544
						Dd-MB-B	MF168545
						Dd-MB-C	MF168546
						Dd-MB-D	MF168547
						Dd-MB-E	MF168548
	Cruz Alta	-61.81850	-33.01805	**C-SF**	Dd.1	Dd-CA-A	MF168549
						Dd-CA-B	MF168550
						Dd-CA-C	MF168551
						Dd-CA-D	MF168552
						Dd-CA-E	MF168553
**Entre Ríos**	Rosario del Tala	-59.08333	-32.31667	**ER**	Dd.10 (U)	Dd-RT-A	MF168464
						Dd-RT-B	MF168465
						Dd-RT-C	MF168466
**Catamarca**	Concepción	-66.05767	-28.65833	**NWA**	Dd.17 (U)	Dd-Cat-A	MF168554
						Dd-Cat-B	MF168555
						Dd-Cat-C	MF168556
**Salta**	Posta de Yatasto	-64.95132	-25.59038	**NWA**	Dd.15 (U)	Dd-Ya-A	MF168557
						Dd-Ya-B	MF168558
						Dd-Ya-C	MF168559
						Dd-Ya2-A	MF168560
						Dd-Ya2-B	MF168561
						Dd-Ya2-C	MF168562
	Metán	-64.98425	-25.47715	**NWA**	Dd.18 (U)	Dd-M-A	MF168563
						Dd-M-B	MF168564
						Dd-M2-D	MF168568
						Dd-M2-E	MF168569
					Dd.30 (U)	Dd-M2-A	MF168565
						Dd-M2-B	MF168566
						Dd-M2-C	MF168567
	Cerro de la Virgen	-65.38118	-24.76473	**NWA**	Dd.22	Dd-CV-A	MF168570
					Dd.31	Dd-CV-B	MF168571
						Dd-CV-C	MF168572
						Dd-CV-D	MF168573
	5 km Virrey Toledo to El Corralito	-65.66562	-24.94223	**NWA**	Dd.32 (U)	Dd-VT-A	MF168574
						Dd-VT-B	MF168575
						Dd-VT-C	MF168576
						Dd-VT-D	MF168577
	Rosario de Lerma	-65.60160	-24.97760	**NWA**	Dd.14	Dd-RL-E	MF168581
						Dd-RL-F	MF168582
					Dd.33	Dd-RL-A	MF168578
						Dd-RL-C	MF168579
						Dd-RL-D	MF168580
						Dd-RL-G	MF168583
	Quebrada de Tilián	-65.53432	-25.12338	**NWA**	Dd.14	Dd-QT-A	MF168584
	Gallinato	-65.37838	-24.66457	**NWA**	Dd.31	Dd-Ga-A	MF168585
						Dd-Ga-B	MF168586
						Dd-Ga-C	MF168587
						Dd-Ga-D	MF168588
						Dd-Ga-E	MF168589
	Bosque Ruta 9 Norte	-65.36585	-24.53122	**NWA**	Dd.22	Dd-R9-A	MF168590
					Dd.33	Dd-R9-B	MF168591

§ column: predefined geographical areas where localities belong (M: Misiones, Cr: Corrientes F: Formosa, Ch: Chaco, ER: Entre Ríos, C-SF: Córdoba-Santa Fe, NWA: Northwestern Argentina).

Unique haplotypes are denoted as (U).

#### DNA extraction, amplification, sequencing and alignment

Genomic DNA was extracted from a single foreleg using CTAB and proteinase K digestion [69]. DNA was purified by phenol ⁄ chloroform extraction and ethanol precipitation [70]. The DNA was resuspended in Tris-EDTA buffer and then stored at -20°C. We amplified a 681-bp fragment of *cytochrome oxidase subunit I* gene (COI), using the universal primers for invertebrates LCO1490 and HCO2198 [71]. Polymerase chain reactions (PCRs) were carried out in a volume of 25 μl including 2.5 μL of 10X core buffer with (NH_4_) SO_4_, 2,5 μL of MgCl_2_ (25mM), 0.25 μL dNTPs (20mM), 0.7 μL of each primer (10μM), 0.15 μL (5U/μL) of Taq DNA polymerase (Fermentas Life Sciences, Brazil), and 1 μL of template DNA (5ng/μL). PCR profile was as follows: initial denaturation at 94°C for 3m, followed by 37 cycles of 94°C for 30s, 48°C for 1m 30s, 72° for 1 min and a final extension at 72°C during 7m. The reactions were performed in an automated thermal cycler Biometra Uno II (Göttingen, Germany). Positive amplifications were purified and sequenced at Macrogen USA Inc (Macrogen Corp. 9700 Great Seneca Highway, Rockville, MD 20850 USA; web: http://www.macrogenusa.com) in an ABI 3730x1 DNA automatic analyzer (PE Applied Biosystems, Forster City, CA, USA). Both forward and reverse sequences were obtained. Sequences were edited using CHROMAS v2.4.1 software (http://technelysium.com.au/). Multiple-sequence alignments were done with Clustal W [72] using the default parameters. This computer-generated alignment was further adjusted manually. All sequences obtained have been deposited in GenBank (accession numbers listed in Table 1).

#### Data analyses and haplotypes

Nucleotide composition, substitution patterns and corrected estimates of sequence divergence were obtained using Kimura’s two-parameter algorithm (K2P) [73], implemented in MEGA 6.06 [74]. The software DnaSP v5 [75] was used to identify the haplotypes in the sequence data set. To better visualize results in maps and trees, haplotypes were identified with color labels according to the geographical areas where the corresponding localities belong (Table 1): Misiones (M, light blue), Corrientes (Cr, red), Formosa (F, brown), Chaco (Ch, purple), Entre Ríos (ER, yellow), Córdoba-Santa Fe (C-SF, green) and NWA (orange). DNA polymorphism was measured by calculating the proportion of segregating sites (S), the haplotype diversity (h) and the nucleotide diversity (π) [76] with Arlequin 3.5 [77] and DnaSP v5.

#### Population structure

To explore the genetic differentiation between sampled localities, the K2P distance was estimated in MEGA 6. The hypothesis of isolation by distance (IBD) was tested by means of a Mantel testin Arlequin 3.5, comparing the pairwise F_ST_ matrix among localities against the corresponding matrix of geographical distances (in km). The significance of the correlation coefficient was assessed by 10,000 permutations.

The presence of population structure in *D*. *dilatatus* was inferred using Bayesian clustering implemented in Geneland [78]. This software estimates different parameters, including the number of populations (K) in a set of georeferenced individuals with sequence data. For this estimate, only individuals from populations with at least five individuals sequenced were considered. First, five independent procedures of 10 x 10^6^ MCMC (Monte Carlo Markov Chain) iterations were run, thinning of 100, burning of 1000 and setting the possible number of populations to 1–10. The uncorrelated model was implemented. Finally, a unique procedure was run with the same parameters as mentioned above, with the K-value set to 4 (the K-value with highest average log posterior probability).

In addition, alternative hypotheses of population structure for *D*. *dilatatus* samples were evaluated through the analysis of molecular variance (AMOVA [79]). The different hypotheses of population structure were defined as follows: 1) one group including all localities; 2) two groups (Mesopotamia vs. NWA), considering the disjunction induced by the sub-xeric Chaco as major barrier (cf. distribution models)[27]; 3) four groups, as indicated by the results of Geneland: Formosa, the rest of Mesopotamian localities, Posta de Yatasto (the most genetically distant locality), and the rest of the NWA.

#### Phylogenetic reconstructions

In a first step, the associations of the COI haplotypes of *D*. *dilatatus* were inferred through the Median Joining Network algorithm [80] using 1000 permutations in the software PopART 1.7 (http://popart.otago.ac.nz). The map of the geographical distribution of haplotypes was obtained with the same software.

The genealogical relationships among haplotypes were assessed using three different phylogenetic approaches: maximum parsimony (MP), maximum likelihood (ML) and Bayesian inference (BI). Ten COI sequences, corresponding to nine gonyleptid species, were used as outgroup. They included: *Geraeocormobius sylvarum* (KM387780.1, selected as root, and KM387832.1), *Pachyloides thorellii* Holmberg, 1878 (GQ912887.1), *Acanthopachylus aculeatus* (Kirby, 1818) (KF726793.1), *Discocyrtus prospicuus* (KR708248.1), *D*. *rarus* B. Soares, 1944 (KR708247.1), *D*. *heteracanthus* Mello-Leitão, 1936 (KR708198.1), *D*. *longicornis* (Mello-Leitão, 1922) (KF726768.1) and *D*. *invalidus* Piza, 1938 (KF726757.1), all acquired from GenBank; in addition, one sequence of *Discocyrtus testudineus* (CDA-F: Puerto Reconquista, Santa Fe Province, GenBank MF168592) was obtained in this study.

Maximum parsimony analysis was executed in PAUP*4.0b10 [81]. Maximum likelihood trees were constructed using the on-line program ‘PHYML with Smart Model Selection’ (available at http://www.atgc-montpellier.fr/phyml-sms/). Bayesian inference was executed in MrBayes 3.2 [82]. Node support for MP and ML trees were evaluated by 1000 bootstrap replicates. For the ML analysis, the ‘General time reversible with proportion of invariant sites and gamma distribution’ (GTR+I+G)[83] was the best model of nucleotide substitution. For BI analyses the best fit model was the GTR+I+G, chosen with jModeltest v2.1.3 [84]. The base frequencies with Dirichlet distribution (freqA = 0.3311, freqC = 0.1826, freqG = 0.0684, freqT = 0.4180), α = 1.0260 and p-inv = 0.4770 were set as priors. To obtain the posterior probability values among clades in MrBayes two independent Markov chain Monte Carlo (MCMC) runs were performed simultaneously, each with one cold and three heated chains. Searches were performed for 10 x 10^6^ generations and sampled every 1000 generations. The first 25% of samples were discarded as a burn-in and the two runs converged on very similar posterior estimates with an average standard deviation of split frequencies of 0.008568. All trees were visualized and edited in FigTree v1.4.2 (available in http://tree.bio.ed.ac.uk/software/figtree/).

#### Divergence time estimates

Divergence times of clades were assessed using BEAST 1.8 [85]. In this analysis, *Discocyrtus invalidus* alone was used as the outgroup, considering its sister-species condition obtained in the precedent analyses. The best fitting model for this data set obtained in jModeltest v2.1.3 was ‘Hasegawa Kishino and Yano’ (HKY +G) [86]. A lognormal relaxed clock was implemented, calibrated with the nucleotide substitution rates estimated by [52] for the Brazilian harvestman genus *Promitobates* (Gonyleptidae, Mitobatinae). This mutation rate was corrected by an order of magnitude for intraspecific analysis, following [87, 88], which resulted in a rate of 0.053 bases/site/Myr. The priors for the molecular clock were normally distributed with a mean equal to 0.053 and a standard deviation of 0.0053. Independent analyses for the following coalescent models were run: constant size, exponential growth and expansion growth. The posterior distributions of parameters were investigated using MCMC analysis, in a total of 100 x 10^6^ steps. The first 10% of resulting trees were discarded as burn-in. The proper mixing of the MCMC search was analyzed in Tracer 1.6 (available at http://beast.bio.edu.ac.uk/Tracer) by calculating the effective sampling size (ESS) for each parameter; ESS higher than 200 indicates convergence of the search. We executed the Bayes Factor test between the three models implemented (constant size vs exponential growth vs expansion growth) to select the best model for our data: ‘expansion growth’. The maximum credibility tree was calculated using Tree Annotator (built into the BEAST package) with a burn-in of 10%. Chronologies for the relevant glacial / interglacial periods (as displayed in the calibrated phylogeny) were based on [89].

#### Demographic history analysis

To evaluate possible events of historical demographic changes in *D*. *dilatatus*, a series of analyses were performed. The statistics D [90] and Fs [91] were calculated to test for departures from the mutation-drift equilibrium, using DnaSP v5.The purpose of these two tests is to distinguish between a DNA sequence evolving neutrally (values not significantly different from zero) or evolving under a non-random process, including directional or balancing selection (significant positive values) or demographic expansion or bottleneck (significantly negative). Fu’s Fs test is particularly sensitive to recent population growth. In addition, the Bayesian skyline plot (BSP) method was implemented to measure the historical changes in effective population size of *D*. *dilatatus*. This analysis was run in BEAST 1.8 on the basis of sequence variation of the COI gene of all individuals of *D*. *dilatatus* included in the study (n = 181); for this data set, the best nucleotide substitution model selected by jModeltest v2.1.3 was the HKY+I model. The analysis was performed using the aforementioned nucleotide substitution rates of *Promitobates* [52]. A lognormal relaxed-clock model was used with a normal distribution mean equaling the substitution rate and a standard deviation of 0.0053. Other settings, including prior distribution, were set as default. Three independent analyses were carried out using five group sizes and posterior distributions of parameters investigated through MCMC analysis in a total of 50 x 10^6^ steps. These three analyses were combined in one chain, with the first 10% of each sample discarded as burn-in, in Log Combiner 1.6.2 (in the BEAST package). The output was analyzed in Tracer 1.6 to ensure a minimum ESS higher than 200 for all sample parameters. The first 10% of the trees were discarded as burn-in to estimate the BSP.

## Results

### Quaternary paleodistributional changes

**Fig 2:**
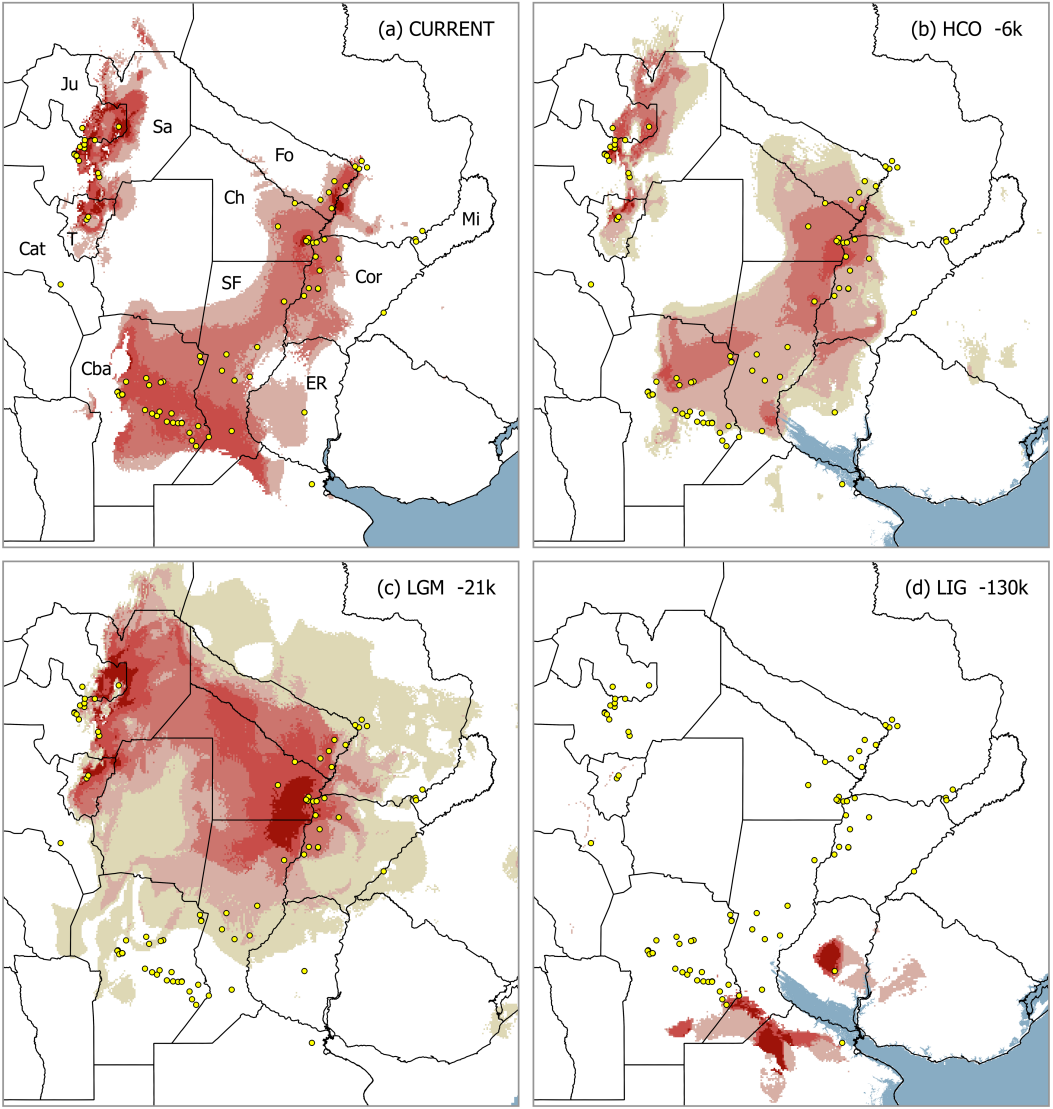
Distribution models of *Discocyrtus dilatatus* for current climate and projection on different Quaternary conditions. (a) Current climate; (b) Holocene climatic optimum (HCO, -6k); (c) Last Glacial Maximum (LGM, -21k); (d) Last inter-glacial (LIG, -130k). Maps (a) and (d) display the mean grid for the 30-replicates run, whereas (b) and (c) represent de averaged grid of the three GCM simulations (CCSM, MIROC and MPI, each one obtained as the mean grid for the 30-replicates run). Scale of suitability (logistic), from light to dark red: 0.4269 (threshold), 0.550, 0.660, 0.760. In the case of (b) and (c), the yellowish area around the prediction shows the maximal span of predictions with all three GCMs together. Training AUC for the 30-replicates run: average 0.969 (0.963–0.972). In maps (b) and (d) the approximate extent of the marine transgressions during -6k (+6 m) and LIG (+9 m) is also displayed. Abbreviations for Argentinean Provinces: Ju: Jujuy, Sa: Salta, T: Tucumán, Cat: Catamarca, Cba: Córdoba, SF: Santa Fe, Ch: Chaco, Fo: Formosa, Mis: Misiones, Cor: Corrientes, ER: Entre Ríos. Map was designed using free spatial data available at http://www.diva-gis.org/Data.

The core range of *D*. *dilatatus* modeled for current climate is remarkably similar to the ‘Mesopotamian opiliogeographical area’, as originally defined [23, 27]: the predicted distribution is widely spread over the Paraná-Paraguay Rivers basin, with a westward projection into the plains of Santa Fe and Córdoba Provinces (Fig 2A). In addition, it depicts the disjunct area along the Yungas piedmont in NWA, thus reflecting the gap across the semi-arid Dry Chaco in the species range. Two estimates of the relative importance of the bioclimatic variables used to build the model (percent contribution, permutation importance) are available in S2 Table.

#### Holocene climatic optimum

The projection onto the HCO layers (-6k) predicted a suitable area roughly similar to that of current climate, somewhat ‘shortened’ in its N-S extension and of lower suitability on average, but without significant displacements (Fig 2B). As for the different GCMs, CCSM resulted the most similar in size to current climate, while ranges obtained with MPI and MIROC were smaller, especially the latter (Table 2). All resultsshow a moderate westward enhancement of the Mesopotamian portion, partially entering into the Chaco gap, though not large enough as to join the NWA sector (Fig 2B). The NWA sector displays some reduction, insinuating the separation of the northern portion (Salta Province) from the southern one (Tucumán Province).

**Table 2:** Changes of range size in *Discocyrtus dilatatus*, as modeled in different chronologies / Global Climate Models (GCM) in the Upper Quaternary.

Chronology	GCM	km^2^	% of current	X-km^2^	% of current	§
Current		435 920.3				
HCO (-6k)	CCSM	437 581.8	100.4			≈
	MIROC	254 294.7	58.3			<
	MPI	372 672.3	85.5			<
LGM (-21k)	CCSM	616 016.6	141.3	*525 505*.*9*	*121*.*0*	>
	MIROC	794 621.4	182.3	*686 401*.*3*	*157*.*5*	>
	MPI	684 702.1	157.1	*630 837*.*4*	*144*.*7*	>
LIG (-130k)		72 065.0	16.5			<<
*Stable area since LGM*		*83 902*.*7*	*19*.*2*			

X-km^2^ column: area estimated for LGM after excluding predicted patches east of 53.4°W. Last column (§) indicates whether range size increased (>), decreased (<) or remained the same (≈), with respect to that predicted under current conditions.

#### Last glacial maximum

**Fig 3:**
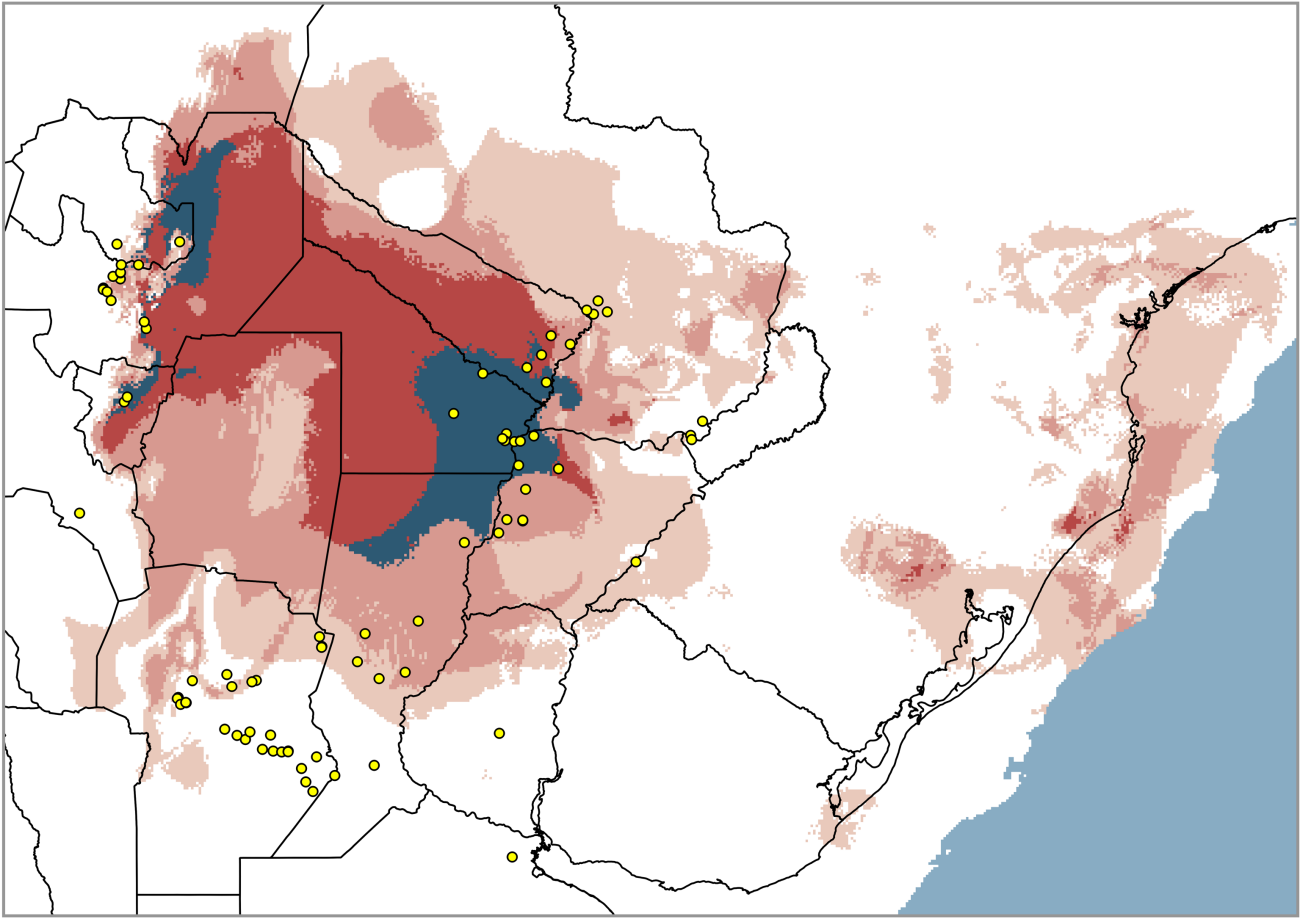
Overlay of the binary predictions of *Discocyrtus dilatatus* for three LGM simulations (CCSM, MIROC and MPI). The overlap area of three, two or one models is displayed in decreasing intensities of red. Within the three-GCM-overlap, the ‘stable area since -21k’ (i.e., the grid-cells shared by all current, -6k and LGM models) is identified with blue.

**Fig 4:**
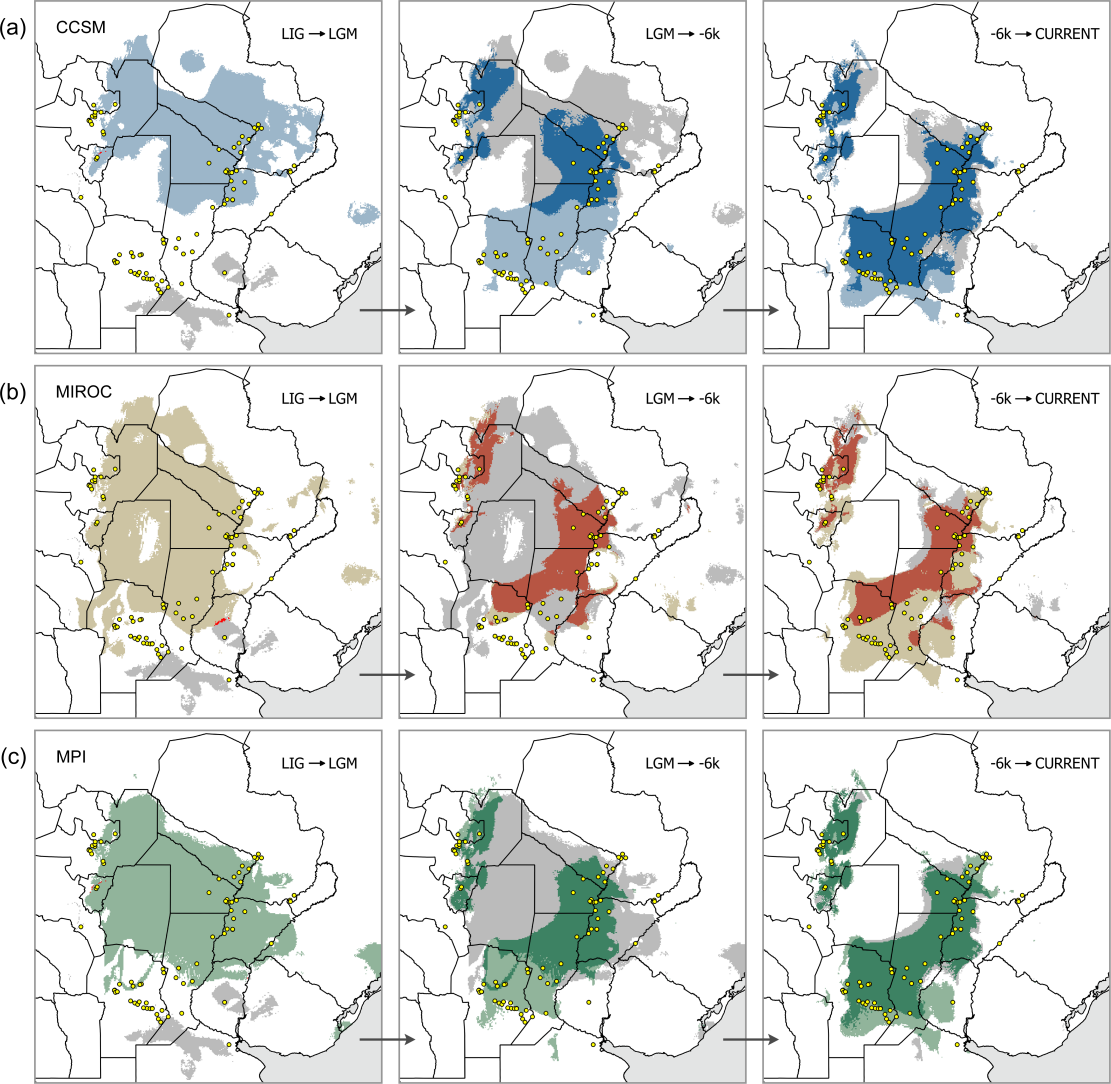
Paleodistributional shifts of *Discocyrtus dilatatus* during the Upper Quaternary, as predicted on three different GCM simulations. (a) CCSM, (b) MIROC and (c) MPI. Grey: areas of the precedent chronology lost during each transition. Color: new range (lighter, areas gained via expansion; darker, areas shared with previous stage). In the case of LIG to LGM transitions, the very few grid-cells shared are in red, to improve contrast. Maps were designed using free spatial data available at http://www.diva-gis.org/Data.

Predictions for -21k displayed remarkable range shifts, and a decided expansion into the Chaco that faded the disjunction (Fig 2C). All three GCM simulations also predicted large, patchy suitable areas around the current Brazilian coast, mostly covering emerged areas nowadays under sea level (Fig 3). In all cases, LGM ranges underwent a considerable increase compared to the current model, even if those eastern patches are clipped out (Table 2). In MIROC the expected northward retreat was moderate, rather entering the Chaco as a broad connection with NWA (Fig 4B). CCSM results were more clear-cut: the range moved almost completely to a wide transverse strip between the ‘Paraguay-Paraná hub’ and NWA, resembling Nores’ paleobridge (Fig 4A). Projection with MPI, broad but ‘bridge-shaped’, was somewhat intermediate. Contrasting to the marked shift predicted by CCSM, both MIROC and MPI models suggest the persistence of suitable areas in Córdoba and Santa Fe Provinces at -21k. Despite their differences, the area shared by all three simulations clearly supports a sort of ‘consensus bridge’ for the LGM (Fig 3).

#### Last interglacial

The greatest changes occurred in the LIG (-130k). In this warming period, the species range reacted with a significant shrinkage and southwards shift (Figs 2D and 4; Table 2): just a small area remained near the southern borders of the current distribution, together with a nearby isolate in Entre Ríos Province and very small patches in NWA.

#### Distributional shifts

The LIG to LGM transition meant a drastic northwards displacement of the suitable area, with negligible overlapping grid-cells recognized as stable, in Tucumán (all models) and Entre Ríos (only MIROC) (Fig 4A–4C). Despite the differences between MIROC, MPI and CCSM, transition from -21k into -6k revealed a roughly similar pattern: the core Mesopotamian portion acquires most of its present shape, and the species range loses the trans-Chaco connection that characterized LGM (Fig 4A–4C). Stable areas of this transition are recognized around the ‘Paraná-Paraguay hub’ (the largest stable portion, Fig 3), extended to Córdoba Province in MIROC and MPI, as well as in NWA. Finally, the ‘-6k to current’ transition was characterized by a moderate shift, in which the borders of suitable areas surrounding the Chaco retracted, and new areas were gained southwards. The current climate appears to have slightly intensified the climatic restrictions in the Chaco, thus reinforcing the gap. In the NWA, this transition enhanced the suitable areas, turning the patchy prediction of -6k into a more continuous strip (Fig 4A–4C).

### Phylogeographic patterns

#### Sequence statistics

A total of 181 specimens from 44 localities were successfully sequenced for the COI gene (Table 1). The final length of analyzed sequences was 681 bp, with no indels detected in the alignments. The analysis of nucleotide sequences showed 60 variable and 27 phylogenetically informative sites. The G-C content was 0.308. The average nucleotide divergence between sequences (K2P) was 1.1%. Table 3 shows some measures of polymorphism in the entire data set and in two partitions (Mesopotamia and NWA). The overall number of haplotypes was 37 and, of those, 30 occurred in the core Mesopotamian sector and nine in NWA (i.e., only two haplotypes were shared by the disjunct areas). Although haplotype diversity (h) was high, the nucleotide diversity (π) was low, as discussed below. The paired differences among sequences (PD) showed that NWA is more heterogeneous than the Mesopotamian sector.

**Table 3:** COI polymorphism in *Discocyrtus dilatatus* for the complete data set and two data partitions.

	N	S	H	h	π	PD	D	F_S_
All samples	181	60	37	0.949	0.01070 +/- 0.00059	7.285820 +/-3.427070	-0.90841	-6.15024
Mesopotamia	143	46	30	0.929	0.00874 +/-0.00039	5.95489 +/-2.857441	-0.86279	-5.2215
NWA	38	35	9	0.893	0.01639 +/- 0.00151	11.160740 +/-5.182104	1.19319	7.14734

N: number of samples; S: average polymorphic segregating sites; H: number of haplotypes; h: haplotypic diversity; π: nucelotide diversity; PD: paired differences among sequences; D and F_S_ = neutrality tests of Tajima (p<0.05) and Fu (p<0.02)

#### Population structure

The pairwise analysis among localities (K2P matrix: S3 Table) showed a remarkable overall similarity, with the exception of Posta de Yatasto (NWA), which is the most genetically distant locality. According to the Mantel test, there is no isolation by distance between localities of *D*. *dilatatus*. The Geneland analysis inferred four genetic clusters of *D*. *dilatatus* in the study area (S1 Fig), of which the most widely extended cluster comprised most Mesopotamian and the northernmost NWA sites (two populations from Salta Province). The remaining three clusters were much more restricted, encompassing: all Formosa localities, the remaining NWA sites, and a single Mesopotamian locality ('Comuna 2 de abril' in Corrientes). The AMOVA analysis resulted in the variance ‘among localities within groups’ always higher than ‘among groups’ and ‘within localities’ (Table 4), demonstrating that neither of the two alternative hypotheses suited to reflect structure (2 and 3) are able to explain the population structure of *D*. *dilatatus*. Hence, historical causes were sought to explain the observed patterns.

**Table 4:** Hierarchical partition of the variance components for haplotypes of *Discocyrtus dilatatus* under different hypotheses.

H	Source of variation	d.f.	Sum of squares	Variance components	% of variation	Φ
1	Among groups	44	515.274	2.66383 Va	72.06	
	Within localities	136	140.45	1.03272 Vb**	27.94	Φ _ST_ = 0.72063**
	Total	180	655.724	3.69655		
2	Among groups	1	26.453	0.22726 Va	5.91	Φ _CT_ = 0.05908
	Among localities within groups	43	488.821	2.58646 Vb**	67.24	Φ _CS_ = 0.71465**
	Within localities	136	140.45	1.03272 Vc**	26.85	Φ _ST_ = 0.73151**
	Total	180	655.724	3.84644		
3	Among groups	3	166.313	1.52635Va**	34.23	Φ _CT_ = 0.34226**
	Among localities within groups	41	348.961	1.90056Vb**	42.62	Φ _CS_ = 0.64793**
	Within localities	136	140.450	1.03272Vc**	23.16	Φ _ST_ = 0.76843**
	Total	180	655.724	3.7607		

Hypotheses (H): (1) one group including all localities; (2) two groups (Mesopotamia vs. NWA); (3) four groups: Formosa vs. rest of Mesopotamian localities vs. Posta de Yatasto vs. rest of the NWA. d.f. = degrees of freedom.

Significance levels of variance and Φ

*p<0.05.

**p<0.01.

#### Haplotype network and geographic distribution

**Fig 5:**
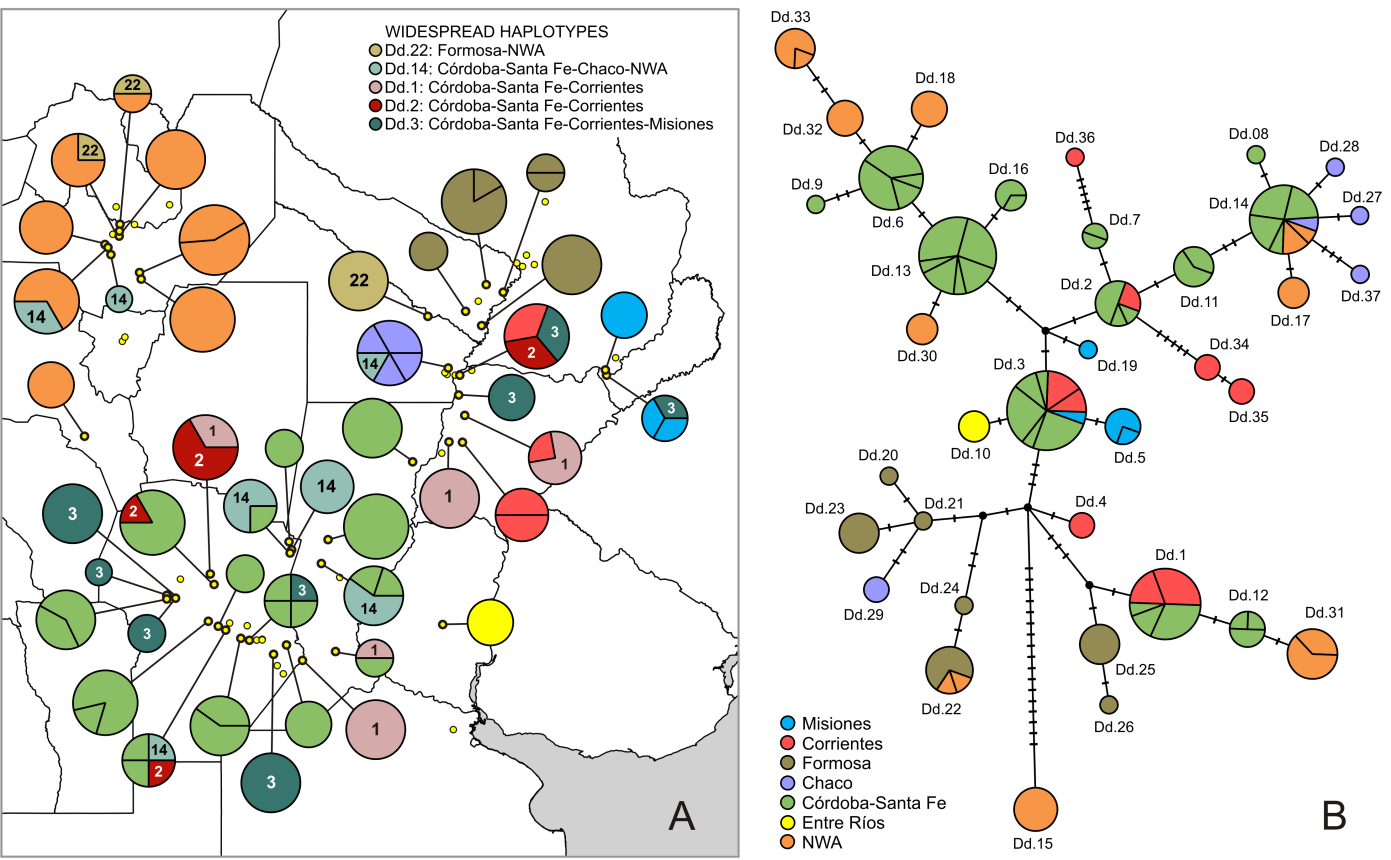
Haplotypes of *Discocyrtus dilatatus*: Distribution and network. (A) Geographical distribution of haplotypes. Circles sizes are proportional to the number of specimens examined in each locality; its internal divisions represent the number of haplotypes per locality. Dots indicate known records of *D*. *dilatatus*; those with thick outline are the localities studied in the phylogeographic analysis. Widespread haplotypes (i.e., shared by two or more sectors of the species range) are identified with a number and the color key in the inset; other haplotypes just use the color standardized in the inset of B. Map was designed using free spatial data available at http://www.diva-gis.org/Data. (B) Median-joining haplotype network. The number of mutational steps between adjacent haplotypes is represented by line marks. The size of circles corresponds to the frequency of each haplotype, and the number of localities where a given haplotype occurs is displayed as internal divisions. Colors indicate the geographical sectors recognized in the species range, as defined in the inset and in Table 1.

The median-joining haplotype network of *D*. *dilatatus* is displayed in Fig 5B. An evident feature of the network is the mostly central position occupied by representatives of the ‘Córdoba-Santa Fe’ sector (green in Fig 5B). The most frequent haplotypes belong to this geographic sector: Dd.13 and Dd.6 (with19 and 13 individuals respectively, all exclusive to ‘Córdoba-Santa Fe’), Dd.3 (20 individuals, of which 5 come from the Corrientes sector and one from Misiones), Dd.1 (16 individuals, of which 8 come from the Corrientes sector) and Dd.14 (15 individuals, of which 3 come from the NWA sector and one from Chaco). The mentioned features may indicate that the ‘Córdoba-Santa Fe’ sector is a main geographic center of diversification for this species. Other sectors of the core Mesopotamian area (Formosa, Chaco, Misiones, Entre Ríos) revealed peripheral positions instead, while, in addition, most of their haplotypes appeared as unique (Table 1). The ‘Corrientes’ sector had a mix of central and peripheral haplotypes (Fig 5B). With respect to the Mesopotamia-NWA disjunction, Dd.14 and Dd.22, quite distant in the network, were the only haplotypes to occur in both sides of the Chaco barrier: Dd.14 shared with localities from ‘Córdoba-Santa Fe’ and ‘Chaco’, Dd.22 with a single ‘Formosa’ site (Fig 5A and 5B). The rest (Dd.15, Dd.17, Dd.18, Dd.30, Dd.31, Dd.32, Dd.33), all restricted to NWA, were placed peripherally in the network, even with some haplotypes exclusive for one locality too (Table 1). It is worth noting that NWA haplotypes joined the network at different sites, i.e., they show independent associations to different haplotypes, mostly from ‘Córdoba-Santa Fe’. In general, genetic distances between Mesopotamian and NWA haplotypes was moderate (between 1 and 5 mutations); as the most relevant exception, haplotype Dd.15, unique to Posta de Yatasto (NWA), was the most divergent of all, separated by 17 mutations from the nearest (hypothetical) node.

#### Phylogeny reconstruction

***Genealogical relationships*.** All three methods of phylogenetic reconstruction placed *D*. *invalidus* as the closest outgroup of *D*. *dilatatus* (S2 Fig). The monophyletic clustering of all *D*. *dilatatus* haplotypes was strongly supported (bootstrap values = 100 for MP, 99 for ML, and a posterior probability of 1 for BI). Haplotype Dd.15 (the most divergent in the network) occupies the basal-most position in MP and ML, as sister group of all the rest. Only for BI this haplotype is positioned more internally in the tree (topologically integrated to the clade named AB4 below, but without support); nevertheless, the BI analysis made for the calibrated phylogeny, under different settings (see below) recovered Dd.15 at the base. As for the rest of the tree, when bootstrap support (MP, ML) or posterior probabilities levels (BI) are strictly considered most branches collapsed, rendering the intraspecific relationships little resolved. Five (with ML and BI) or eight (with MP) individual haplotypes join the tree in a large sub-basal polytomy. The same polytomy links a number of discrete internal lineages, quite constant across different methods (they are recognized, in full or partially, by MP, ML and BI). Those clades show evidence of a rough correspondence to the geographic sectors recognized in *D*. *dilatatus*, although some of them are indeed somewhat mixed. They include the following (S2 Fig):

- *Clade A*: six haplotypes almost restricted to the ‘Formosa’ sector, except for one haplotype from an adjacent ‘Chaco’ site, and Dd.22, shared by Formosa and NWA localities. This clade is only supported in MP.
- *Clade B*: more heterogeneous, it comprises two subclades: one from the ‘Formosa’ sector, and another one with haplotypes from ‘Córdoba-Santa Fe’, ‘Corrientes’ and NWA.
- *Clade AB4*: clade A, clade B and haplotype Dd.4 (‘Corrientes’) form a more comprehensive cluster in MP. In ML and BI, these clades are still linked by topology, but without support. As already mentioned, for BI this cluster includes Dd.15, otherwise basal and isolated.
- *Clade C*: with majority of haplotypes from the ‘Chaco’ sector, except for Dd.8 (‘Córdoba-Santa Fe’), Dd.17 (NWA) and Dd.14, shared by all three mentioned sectors. With BI, Dd.11 (from ‘Córdoba-Santa Fe’) joins clade C basally to form the higher-level clade C11 (not recovered in MP, and only recovered by topology in ML).
- *Clade D*: a small group of two ‘Corrientes’ haplotypes, identified by all three methods.
- *Clade E*: also a two-haplotypes clade, combining ‘Corrientes’ and ‘Córdoba-Santa Fe’ sectors, only strictly retrieved by BI (ML recovered this cluster without support).
- *Clade F*: a large cluster formed by four ‘Córdoba-Santa Fe’ and four NWA haplotypes.

It should be noted that although the ML tree is displayed fully resolved, nodes with bootstrap support < 50 are considered unsupported, thus treated as collapsed in the preceding description. For BI, the cut-off value is 0.67.

**Fig 6:**
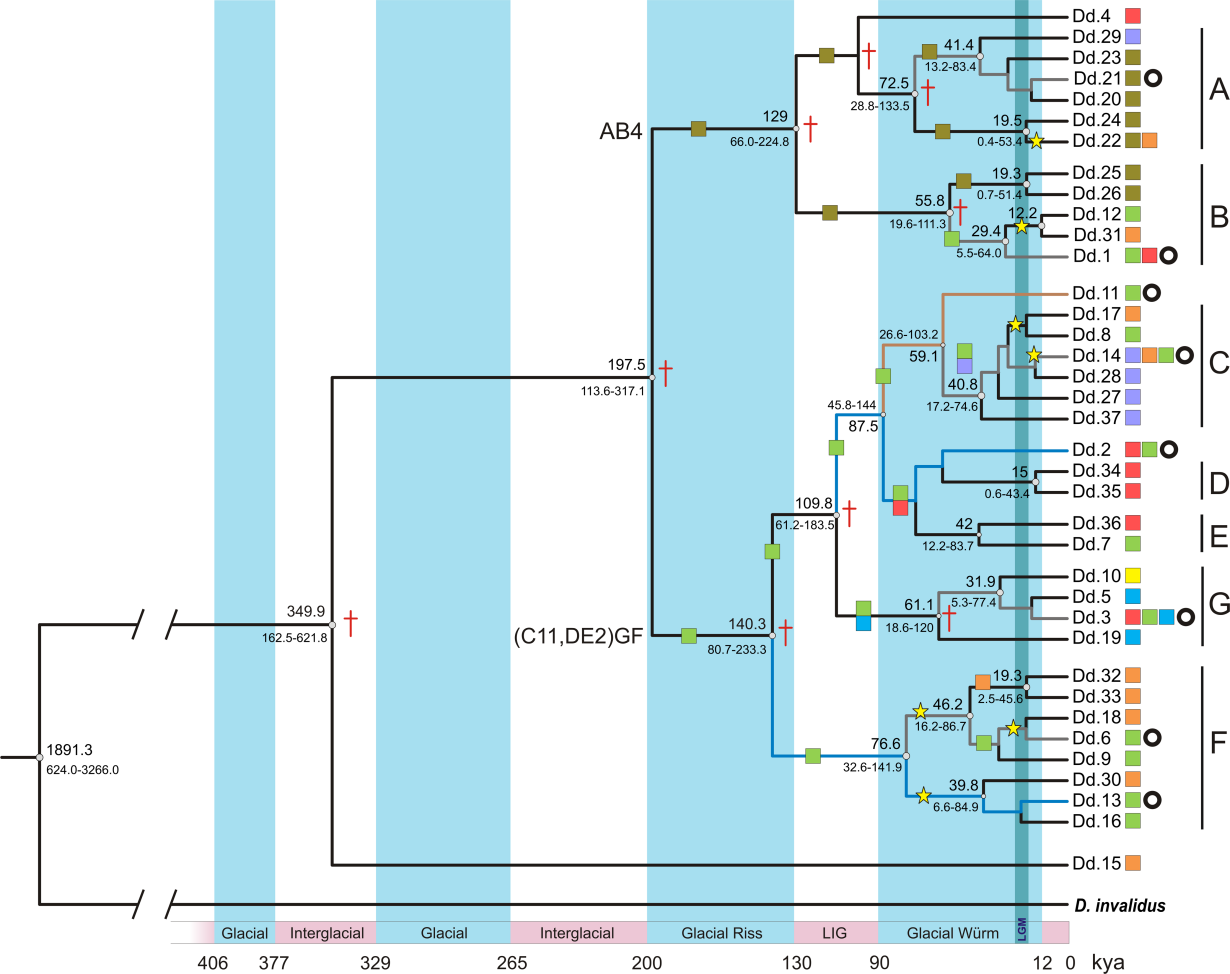
Calibrated maximum-clade-credibility tree obtained with beast. Estimated ages (large numbers) are given only for nodes with posterior probability >0.67 (indicated with open dots; the higher support, the larger dots); small numbers display the 95% credibility interval. Supported clades are identified by letters (A to G). Color squares refer to the geographical areas predefined in Fig 5, and circles point out the ancestral haplotypes, as recognized by their position in the median-joining network; their chronological span in the tree is indicated with a distinct color in branches (grey, blue, brown). Stars indicate presumed expansion events affecting NWA haplotypes. Crosses at some nodes display the position in the phylogeny of the hypothetical nodes recovered in the haplotype network. Pleistocene glacial-interglacial cycles, with ages in kya, are represented by vertical stripes; Last Glacial Maximum (LGM) in darker blue.

***Divergence times*.** The same main clades were retrieved in the calibrated phylogeny (Fig 6).This tree resulted in an identical topology as the ML phylogeny (S2 Fig), with the advantage that some previously unsupported dichotomies gained support. The earliest divergence event is that of haplotype Dd.15 (Posta de Yatasto, NWA), dated to the interglacial at about 350 kya. The second cladogenesis is identified at 197.5 kya, representing the origin of two major lineages of *D*. *dilatatus*: on the one hand, AB4 (now supported, primarily associated to the ‘Formosa’ sector), and on the other hand, an heterogeneous assemblage of ‘Córdoba-Santa Fe’, ‘Corrientes’, ‘Chaco’ and related haplotypes: clades C+Dd.11, D, E, together with Dd.2, the newly recognized clade G, and clade F (i.e., ‘(C11,DE2)GF’ in Fig 6). Clade G reunites three peripheral haplotypes (Misiones, Entre Ríos) and the widespread Dd.3 (Córdoba, Corrientes, Misiones), all consistently collapsed in the previous MP, ML or BI analyses. Each major clade (‘AB4’ and ‘(C11,DE2)GF’) underwent several divergence events during the following glacial (Riss) and interglacial (LIG) cycle. It is in the Last Glacial period (Würm glaciation) when most NWA haplotypes arise from independent Mesopotamian lineages (Fig 6), namely:

- Dd.22 (clade A), shared by NWA and ‘Formosa’, might have expanded around 19.5 kya.
- Dd.31 (clade B): segregates from Dd.12 (‘Córdoba-Santa Fe’) at the start of Holocene (12.2. kya), but its ancestral Mesopotamian lineage might have expanded between 29.4 and the referred date.
- In clade C, separation of Dd.17 from Dd.8 (‘Córdoba-Santa Fe’) does not have support, although the tree displays a possible expansion around LGM and divergence shortly thereafter. In a closely neighboring branch, Dd.14 is shared by NWA, ‘Chaco’ and ‘Córdoba-Santa Fe’, also with a presumed expansion (unsupported) after the LGM. The earliest supported date retrieved for this whole lineage is about 40.8 kya, in middle of Würm glacial.
- Clade F: this lineage contains NWA and ‘Córdoba-Santa Fe’ haplotypes, with several divergence events occurring along the Würm glacial. The earliest divergence is recognized at 76.6 kya, differentiating two ‘ancestral’ haplotypes from ‘Córdoba-Santa Fe’: Dd.6 and Dd.13. In turn, each one gave rise to independent NWA lineages: Dd.6 originated Dd.32 + Dd.33 by 46.2 kya and Dd.18 sometime around LGM; Dd.30 emerged from Dd.13 at 39.8 kya.

#### Demographic history of *D*. *dilatatus*

**Fig 7:**
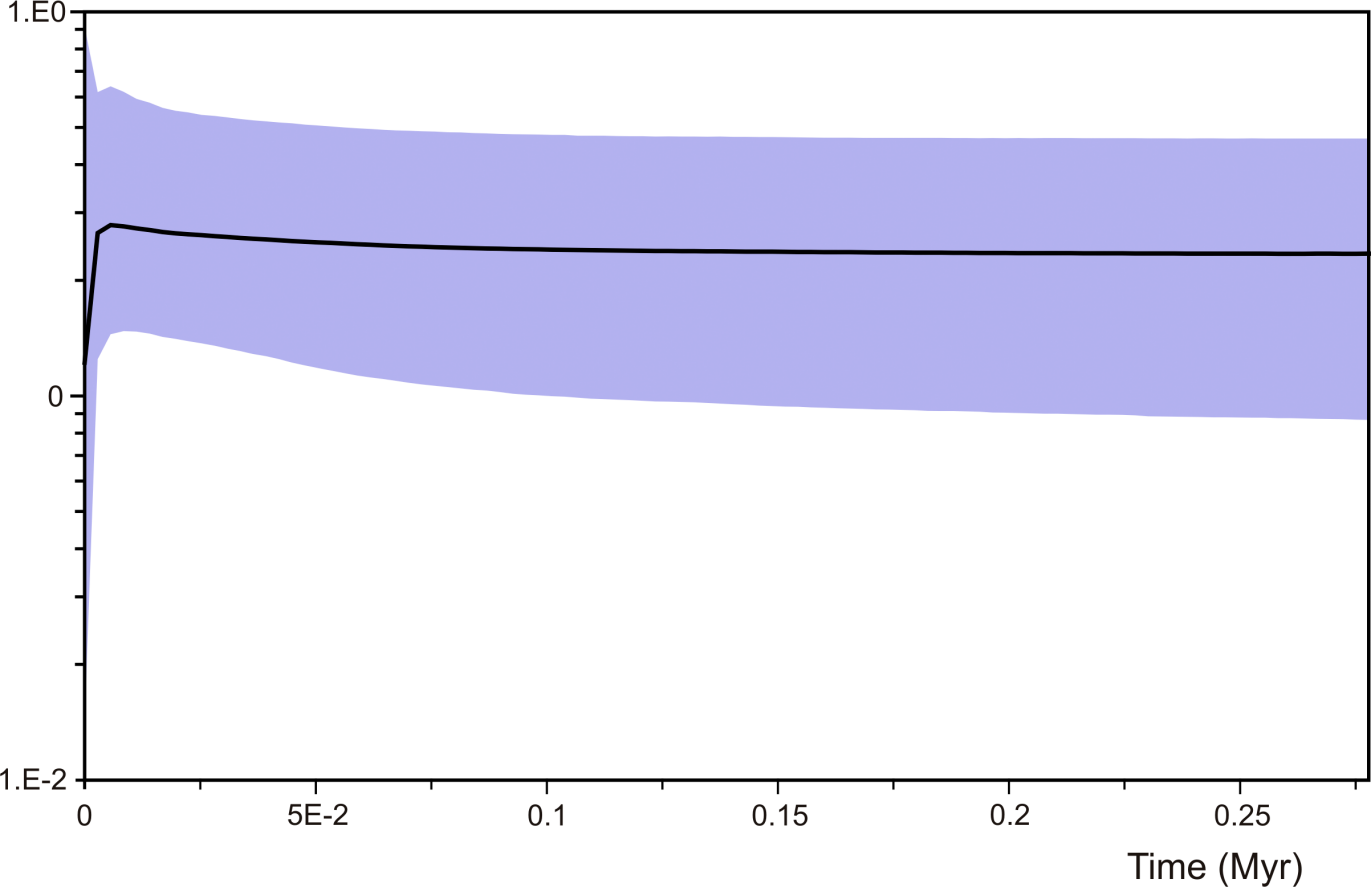
Bayesian skyline plot, depicting the demographic history of *Discocyrtus dilatatus*. The y axis represents the increase of the effective population size and the x axis represents the time in million years. The solid line indicates the mean value of the population size over time; the shaded area displays the 95% confidence interval.

The results of the Tajima's and Fu's test are shown in Table 3. For both tests, the three partitions of the data set were non-significant, indicating that populations of *D*. *dilatatus* did not experience sudden changes in size. However, these results are in contradiction to those obtained by the BSP analysis (Fig 7), where the plot indicates that the species, after a long period of smooth, slight increase of the population size, is currently going through an exponential decrease. This abrupt decline started very recently, at around 0.6 kya.

## Discussion

The combined evidence obtained from two independent sources (paleodistributional reconstruction and phylogeographic analysis) clearly supports the historical origin of NWA populations of *D*. *dilatatus* caused by range expansion / retraction cycles in the Quaternary. This is the first case in which biogeographic evidence was found in favor of the historical explanation for the disjunct occurrence of a Mesopotamian harvestman in NWA. It is in clear contrast with the Paranense species *Geraeocormobius sylvarum*, for which, as stated, recent anthropic introduction into NWA was inferred [24, 33, 34]. Although the preceding statements sound similar to Nores’ hypothesis [15, 16] (the LGM-CCSM reconstruction and the ‘consensus bridge’ of Fig 3 have an astonishing resemblance to Nores’ paleobridge), the historical scenarios obtained in the present research appear more complex. More importantly, they differ from the original postulate in some significant aspects, as discussed below.

### Expansion recovered in glacial stages

While the formation of the paleobridge was attributed by Nores [15, 16] to one or several interglacial periods (warm-humid), our results indicate that the range of *D*. *dilatatus* expanded across the inhospitable Dry Chaco in the LGM. This expansion + paleobridge scenario is supported by all three LGM models, but only CCSM combined this effect with a marked northward shift (as expected). Additional areas are predicted for all GCM along the Brazilian coast (and over the current continental shelf, emerged at those times: Fig 3), although there is no evidence (and it seems indeed doubtful) that the species effectively occupied those distant suitable patches. Researchers agree that climate deterioration during LGM led to a global cooling of -4.5°C to -5°C, and to 30% less precipitation than today [6, 9, 92]. On the contrary, LIG (as the warmest time slice examined) and HCO (the amelioration stage that followed LGM) reinforced the disjunction. To understand this apparent contradiction–for harvestmen–one should take a look into the ecological constraints underpinning the current disjunction: it seems not to be drought, but rather the extreme temperatures, that inhibits the range expansion of *D*. *dilatatus* and other Mesopotamian harvestmen into the Chaco [26, 27, 38]. In particular, the variable ‘bc5-maximum temperature of warmest month’, which undoubtedly depicts the mentioned harsh conditions the best, was identified as the most relevant current ‘limiting factor’ along the westernmost margins of the core Mesopotamian range (i.e., those bordering the Dry Chaco) [26, 27]. Comparison of bc5 cumulative frequency curves suggested a similar ‘abrupt’ boundary of maximal temperature for three Mesopotamian species, *D*. *dilatatus*, *D*. *testudineus* and *G*. *orensis*, not crossed beyond 34.40°C–34.60°C [38]. It is easy to infer that LGM conditions likely broke down the climatic barriers through the attenuation of those limiting temperatures. This might have happened either along a narrow suitable strip or on a broad contact area (Figs 2C, 3 and 4A–4C).

This interpretation received strong support in the molecular analyses: most of the several expansion events from the Mesopotamia to NWA were recovered by the dated phylogeny either during LGM, or, more vaguely, sometime in the last glacial period (Fig 6). The phylogeographic results provided many details of the disjunction history that were not contained in the paleoclimate SDMs. As observed, with the exception of Dd.15 (discussed below), NWA haplotypes are attached to the network at separate, peripheral positions, and connect to different Mesopotamian haplotypes by few mutational steps (in most cases, from the ‘Córdoba-Santa Fe’ sector). In other words, although they belong to independent Mesopotamian lineages, most appear to have undergone expansive/vicariant events almost synchronously, around the LGM. This might indicate that a single paleoclimatic event that drove the expansion at -21k, followed by vicariance and segregation shortly thereafter, would be enough to explain the rise of most NWA haplotypes: several independent Mesopotamian lineages might have taken advantage of the same suitable ‘climatic bridge’ to cross the Chaco. Just two NWA haplotypes did not complete their segregation, and are still shared with their Mesopotamian source localities. In the case of clade F, the expansion/vicariance process appears to have started somewhat earlier, still all within the last (Würm) glacial period. Haplotype Dd.15, unique to Posta de Yatasto (NWA), may represent a special situation, as revealed by the molecular evidence alone. It is peripheral in the network too, but is linked to the rest by the highest genetic distance; moreover, its nearest connection is not with an existing haplotype but to a hypothetical node (Fig 5B), strongly suggesting a relictual condition. This feature is also reflected in most phylogenies, in which Dd.15 is the basalmost diverging haplotype of *D*. *dilatatus*. Extrapolating the precedent interpretation for LGM, it can be speculated that Dd.15 is the result of an older expansion event, likely during one glacial stage (406–377 kya?), to diverge at around 350 kya, i.e., during one interglacial period. To summarize, there is evidence that expansion into NWA would have happened at least twice in glacial times during the Upper Pleistocene, followed by segregation of most isolates. No trace of comparable events was detected for the two glacial stages in between (329–265 kya, and 200–130 kya = Riss).

### What happened in the interglacials

After the last divergence events, between the end of the Würm period and the beginning of the Holocene, all lineages remained unchanged. At this stage, the return to higher maximal temperatures started to dismantle the LGM paleobridge, leading to a range during the mid-Holocene (-6k) that was similar to the current disjunct pattern (Fig 2A and 2B). The general temperatures of -6k resembled those of present day, only slightly cooler (global annual cooling less than -0.1°C), with more significant changes recorded regionally and seasonally [92]. This moderate cooling may explain why the -6k models show a slight expansion into the Dry Chaco, still maintaining the disjunction.

Effects of global warming during LIG (-130k) proved to be the most drastic on the distribution of *D*. *dilatatus* (Fig 2D). The range was strongly reduced and displaced southwards, with main portions remaining in ‘Entre Ríos’ and south of the current ‘Córdoba-Santa Fe’ sector, along with a few minor marginal patches in NWA. According to the dated phylogeny (Fig 6), there were two major COI lineages evolving during LIG: one, ‘(C11,DE2)GF’, primarily referring to the ‘Córdoba-Santa Fe’ sector, and the second one (AB4) associated with the present ‘Formosa’ sector. Those lineages, differentiated near the boundary between Riss (penultimate) glacial and the preceding interglacial, experienced further internal diversification during LIG and the following Würm (last) glacial stage. However, while the ‘Córdoba-Santa Fe’ lineage could be assigned to the main southern patch retrieved by LIG models, SDMs seemingly failed to predict a patch where the ‘Formosa’ lineage must undoubtedly have persisted. Indeed, SDMs are ‘coarse scale’ models so that many local landscape conditions can be left undetected; and the ‘Formosa’ lineage could have well survived in small remains of gallery forest associated to wetlands within the huge alluvial fans of the Bermejo or Pilcomayo Rivers [93]. All present ‘Formosa’ localities are placed north of Bermejo River (Fig 5); as a major geographical feature, it is deemed to have favored the initial isolation of AB4, until ulterior expansion into other areas was possible (Fig 6). No haplotype from other clades was detected north of this river.

### Ancestral haplotypes

Some haplotypes with a central position in the network are interpreted as ‘ancestral-still-existing’ to a given lineage. They can be identified and dated through the combined inspection of the network and the calibrated phylogeny. Most of them cannot be traced back beyond the Würm (last) glacial stage (Fig 6): Dd.21 (clade A) up to 72.5 kya; Dd.1 (clade B), up to 55.8 kya; Dd.14 (clade C), 59.1 kya; Dd.11 (clade C11, likely ancestral to Dd.14), 87.5 kya; Dd.3 (clade G), 61.1 kya; and Dd.6 (clade F), derived from Dd.13 at 76.6 kya. With the same rationale, only two ancestral haplotypes appear to be older: Dd.2 (no major clade; from which C11, D and E originate), likely arose at 109.8 kya (during LIG); Dd.13 (clade F), from which Dd.6 was derived, can be tracked as early as 140.3 kya, i.e., in the Riss (penultimate) glacial. The median vectors or hypothetical nodes generated by the software to join the parts of the network are also transferable to the calibrated tree, with the meaning of either extinct ancestors or simply missing (not yet discovered) haplotypes. The BSP analysis (Fig 7) shows an important reduction in the effective population size in the last 0.6 kya, perhaps associated to the extinction of central haplotypes, maybe due to a bottleneck effect. When a population bottleneck occurs, the extinction of new haplotypes (tips) might not leave an evident genetic footprint, but the extinction of ancestral haplotypes would generate genetic gaps (the hypothetical nodes in the network).

### Geographical mixing of lineages

Populations of *D*. *dilatatus* are characterized by a low nucleotide diversity (mean π = 0.01070) and the lowest overall COI divergence hitherto reported for harvestmen (1.1%) [50, 51], accompanied by a high haplotype diversity (h = 0.949). These combined features suggest a high dispersion for this species (as attributed to *Aoraki denticulata major* [48]), which might have facilitated expansion whenever environmental conditions became suitable. The expansion episodes might have allowed not only the connection to NWA, but also internal interchanges among Mesopotamian sectors, especially in contiguous localities, contributing to the current mixed composition of most phylogenetic lineages. ‘Córdoba-Santa Fe’ seems the most dynamic sector in such long distance north- and southwards cyclic displacements, hence its central role in the intraspecific diversification history. Several localities on the southernmost borders of ‘Córdoba-Santa Fe’ contain some unique or restricted haplotypes (Dd.7, Dd.8, Dd.9, Dd.11, Dd.12; Table 1) that might represent the remains of those expansion-retraction cycles across the plains.

### Did rivers act as barriers?

Together with paleoclimate changes, which in this paper are focused as the main environmental drivers of range shifts, also geomorphological features might have played a role by offering some specific constraints to dispersal. The global scenery consisted of an extensive alluvial basin, which remained stable since at least 1 Mya. But the region is dominated by a complex and changing hydrological system, which probably had some impact on particular lineages; its precise correlation with the described scenarios, however, remains largely conjectural. Like other South American sedimentary basins, the Paraguay-Paraná plains suffered several events of marine transgressions along its geological history [94]. The very last, large-scale ingression affecting the whole region took place in the ‘Aftonian’ interglacial (referable to MIS 22, 1.3–0.9 Mya), in which sea rose to ~76–100 m above present level [95, 96]. This transgression must have flooded most of the area now occupied by *D*. *dilatatus*, leaving only Córdoba and NWA emerged, but no signal of this event can be traced within our available evidence (Fig 6). All subsequent Quaternary ingressions were much weaker, their influence being limited to the lower Paraná basin (Fig 2B and 2D). To which extent this river worked (or works) as a barrier to dispersal is not well understood, but as a fact, a number of haplotypes are more linked to the spread into the eastern side (still somewhat mixed), as can be inferred for clades G and ‘DE+Dd.2’ (Fig 6). None of these clades is involved in the NWA connection, but they represent a different eastern expansion pattern instead, developed in the Last Glacial (Würm) as well. Clade G is composed by Dd.3, widespread in ‘Córdoba-Santa Fe’, ‘Corrientes’ and ‘Misiones’, and the marginal haplotypes Dd.5 and Dd.19 (‘Misiones’) and Dd.10 (‘Entre Rios’). Arisen in the LIG (MIS 5e) interglacial (Fig 6), clade G could be associated to the ‘Entre Ríos’ patch predicted at this stage (Fig 2D). During most of MIS 5e, sea was +3 to 4 m above present level, with a brief highstand of +6 to 9 m at the end of the stage (-120 k) [97, 98]. While this sea level rise produced just a modest transgression into the lower Paraná basin (Fig 2D), it probably reinforced the initial independence of clade G, by keeping the two patches modeled in LIG separate. In this scenario, clade G might have shifted northwards during LGM; it is not clear, however, whether its presence in ‘Córdoba-Santa Fe’ reveals a secondary expansion (Fig 6). As for ‘DE+Dd.2’, the ancestral (and widespread) haplotype Dd.2 is shared by ‘Córdoba-Santa Fe’ and ‘Corrientes’, while its derivatives are limited to either one of these sectors. The ‘Corrientes’ haplotypes gather in the NW corner of this province, an area where the steady northward drift of the Paraná River up to the end of LIG resulted in an actively changing landscape [99]. This process shaped a net of diverging diagonal riverbeds that dissected the area (the ‘Paraná alluvial mega-fan’ [93]), much likely splitting the species range more than once. Interestingly, the ‘Corrientes’ haplotypes appear ‘scattered’ on the network (Fig 5B), as if they were the result of some kind of fragmentation event. The current genetic diversity of a genus of fossorial rodents inhabiting the area was explained by the referred patchy, unstable environment too [100].

### Alternative hypotheses for the disjunction

It is worth mentioning that papers dealing with sub-Andean–Paranense disjunctions in plants appeal to a missing corridor too, but they typically suggest a different location for it. Those studies postulate a bridge across northern Paraguay and southeastern Bolivia, as a part of a putative ‘Pleistocenic arc’ that joined together patches of ‘Seasonally Dry Tropical Forests’, currently separated, during glacial maxima [18, 101, 102] (but see [13] for opposing hypotheses). A similar connection was insinuated by [103] in a criticism to Nores’ hypothesis. Moreover, in a recent study, combining SDM and phylogeographic analyses of one disjunct bird species, a trans-Cerrado connection during LGM (which can be assimilated to the ‘Pleistocenic arc’) was supported as the main migration route linking both sides of the disjunction [17]. In the same paper, Nores’ paleobridge was explicitly evaluated as an alternative hypothesis, resulting in weak and inconclusive evidence for a presumed trans-Chaco connection during LIG, only based on SDM projections. In the absence of molecular evidence, these results should be taken with caution: they may only reflect the climatic suitability of the modeled bird species, but they tell us nothing about the probability that forests (as a crucial requisite) really expanded in that period. Meanwhile, the possible role of the ‘Pleistocenic arc’ as an alternative dispersal route for *D*. *dilatatus* did not receive any support in the present study. In fact, except for an isolated finding of *Gryne orensis* in the Brazilian Pantanal [26], no Mesopotamian harvestman species has hitherto been recorded so far north.

### Concluding remarks

Table 5 summarizes the major biogeographic events identified in this research. These results offer an improved hypothesis to explain the disjunction by means of an ephemeral past connectivity. They also change a previous paradigm, that of treating the LGM as a retraction stage: for *D*. *dilatatus*, at least, it was just the opposite. Whereas Paranense birds of Nores’ hypothesis depend on forests and would need a real ‘forest bridge’ as an expansion route, independent evidence for the existence of such a corridor during the Pleistocene is lacking (no palynological record is available for the Pilcomayo and Bermejo basins area [104]). But this forest prerequisite is likely not as strict for *D*. *dilatatus* and other Mesopotamian harvestmen, and just the continuity of suitable conditions (say, a ‘climatic bridge’), eventually favored by gallery vegetation along riverbanks, would have been enough to enable expansion. The combined SDM/molecular analyses available thus far for harvestmen with disjunct ranges (*D*. *dilatatus*, *G*. *sylvarum*) demonstrate that responses to climate change are largely species-specific; these should be further investigated individually before sound generalizations can be achieved.

**Table 5:** Major biogeographical scenarios proposed for the Upper Quaternary history of *Discocyrtus dilatatus*, based on the integrated molecular and paleodistributional evidence.

~kya	Molecular evidence	Paleodistribution	Remarks
1890	Divergence from *Discocyrtus invalidus*.	N/A	
350	Early divergence of Dd.15 (Posta de Yatasto) from all the rest.	N/A	Hap.15 is considered relictual, as evidence for the oldest recoverable event of formation of a Mesopotamia-NWA paleobridge (likely in the precedent glacial stage, 406–377 kya?), followed by isolation in the 377–329 kya interglacial.
198	First major divergence of lineage AB4 (dominant in Formosa sector) from the rest.	N/A	This divergence might be associated with the end of the interglacial stage at 265–200 kya, or the beginning of the following glacial (Riss).
130	Divergence events in the Formosa clade; divergence event in the Mesopotamia clade.	LIG or MIS 5e. Range strongly reduced and displaced to the south, due to global warming.	Molecular evidence supports the persistence of the Formosa clade (formed in the previous interglacial) and the Hap.15 in NWA; paleodistributional models were unable to detect such persisting remains.
90–12	The immediate ancestor of NWA haplotypes, in several independent lineages, as well as the segregation events, can be recognized along the last glacial period (Würm), in many cases around LGM (-21k).	LGM (cooling stage). CCSM, MIROC and MPI simulations predict the connectivity between the core Mesopotamian sector and NWA, because of the lowering of maximal temperatures in the Chaco.	This stage is interpreted as an expansion period, in which independent, pre-existing lineages took advantage of the paleobridge (climatic suitability) formed at LGM. As the connection faded, haplotypes were left isolated in NWA (in most cases with haplotype differentiation by the end of LGM).
6	No divergence event was recognized in the phylogenetic tree at this stage.	HCO. Connectivity between Mesopotamia and NWA disappears.	Stage of *in situ* retraction of the Mesopotamian sector. It will be followed in current climate by a moderate southward expansion and the strengthening of the disjunction.

## Supporting information

S1 File Coordinates of effective records used to model *Discocyrtus dilatatus* (Maxent.csv file). (CSV)

S1 Table AICc (‘corrected Akaike information criteria’) scores obtained in the tuning experiment run to determine the MaxEnt ‘best model’ parameters for *Discocyrtus dilatatus*. (DOCX)

S2 Table Relative importance of the 10 bioclimatic (bc) variables used to model the distribution of *Discocyrtus dilatatus*. (DOCX)

S3 Table Pairwise Kimura 2 parameters (K2P) distance between localities of *Discocyrtus dilatatus*. The highest values are highlighted.
(XLS)

S1 Fig Map of population membership from a Bayesian population analysis in Geneland. The different colors indicate the hypothetical populations (four in this case) inferred by the analyses.
(DOCX)

S2 Fig Genealogical relationships of haplotypes of *Discocyrtus dilatatus*. (A) Maximum likelihood (ML), (B) Maximum parsimony (MP), and (C) Bayesian Inference (BI). For each tree, nodes are given the corresponding statistical support value, if applicable (bootstrap over 50 for ML and MP, 1000 pseudoreplicates; posterior probability over 0.67 for BI). In (A) ML phylogeny, the support values for all three methods are given (ML bootstrap/MP bootstrap/BI posterior probability).
(DOCX)
