# Supplementary material for: The disjunct pattern of the Neotropical harvestman Discocyrtus dilatatus (Gonyleptidae) explained by climate-driven range shifts in the Quaternary: Paleodistributional and molecular evidence
Source: PLoS One. 2017 Nov 15;12(11):e0187983. doi: 10.1371/journal.pone.0187983 (PMC5687770; doi:10.1371/journal.pone.0187983)
Supplement: S1 Table — (DOCX) [file pone.0187983.s002.docx]

The disjunct pattern of the Neotropical harvestman *Discocyrtus dilatatus* (Gonyleptidae) explained by climate-driven range shifts in the Quaternary: paleodistributional and molecular evidence

**S1 Table:** AICc (‘corrected Akaike information criteria’) scores obtained in the tuning experiment run to determine the MaxEnt ‘best model’ parameters for *Discocyrtus dilatatus.*

Scores result from the comparison of 85 possible outputs, derived from 17 combinations of linear (L), quadratic (Q), product (P), threshold (T) and hinge (H) features, and regularization multiplier 1 to 5. L+H combination was not considered to avoid redundancy. The ‘best model’ corresponds to the lowest AICc value (QT-1 in the first table).

**Below:** second-round analysis to refine the tuning by incorporating intermediate regularization multipliers between 1 and 2 (the latter, obtained by Morales et al. 2017 for an almost identical sample). Final best model: QT-1.25.

|  | **Regularization multiplier** | | | | |
| --- | --- | --- | --- | --- | --- |
| **FEATURES** | 1 | 2 | 3 | 4 | 5 |
| LQ | 1762.1358 | 1781.7689 | 1787.4578 | 1795.2187 | 1807.7311 |
| LP | 1758.7127 | 1788.1756 | 1790.7176 | 1795.7154 | 1806.4361 |
| LT | 1719.7074 | 1722.1600 | 1728.3519 | 1744.3322 | 1761.0732 |
| LQP | 1738.1759 | 1764.9438 | 1776.9549 | 1792.4561 | 1802.2111 |
| LQT | 1718.1177 | 1732.7214 | 1740.2950 | 1746.4087 | 1763.3053 |
| LPT | 1789.4074 | 1730.4528 | 1731.1885 | 1752.8570 | 1772.4761 |
| LQPT | 1778.9409 | 1743.5073 | 1746.1572 | 1753.9825 | 1773.9148 |
| QP | 1734.9218 | 1764.7131 | 1776.8116 | 1792.2464 | 1802.1844 |
| QH | 1750.1628 | 1763.5779 | 1764.4131 | 1773.2369 | 1774.9197 |
| QT | **1714.1858** | 1719.8889 | 1731.7804 | 1738.3965 | 1754.1457 |
| QPH | 1749.2066 | 1758.3839 | 1765.7716 | 1777.8778 | 1791.1693 |
| QPT | 1778.9409 | 1743.6429 | 1738.5589 | 1753.8728 | 1773.9994 |
| QHT | 1801.5679 | 1734.6878 | 1745.7703 | 1753.5452 | 1756.5893 |
| QPHT | 1805.7965 | 1766.4941 | 1742.4606 | 1753.2378 | 1766.8053 |
| PH | 1756.0395 | 1746.1023 | 1771.2925 | 1787.2181 | 1797.3141 |
| PT | 1789.4074 | 1730.4528 | 1731.1056 | 1749.5298 | 1772.2978 |
| HT | 1791.7533 | 1751.3991 | 1752.1350 | 1749.8463 | 1750.8465 |
|  |  |  |  |  |  |
| Round 2 | **Regularization multiplier** | | | | |
| **FEATURES** | 1.00 | 1.25 | 1.50 | 1.75 | 2.00 |
| **QT** | 1714.1858 | **1704.7271** | 1708.5817 | 1718.6651 | 1719.8889 |

Reference:

Morales NS, Fernández IC, Baca-González V. MaxEnt's parameter configuration and small samples: are we paying attention to recommendations? A systematic review. PeerJ. 2017; 5:e3093. doi: 10.7717/peerj.3093
