# Supplementary material for: The disjunct pattern of the Neotropical harvestman Discocyrtus dilatatus (Gonyleptidae) explained by climate-driven range shifts in the Quaternary: Paleodistributional and molecular evidence
Source: PLoS One. 2017 Nov 15;12(11):e0187983. doi: 10.1371/journal.pone.0187983 (PMC5687770; doi:10.1371/journal.pone.0187983)
Supplement: S2 Table — (DOCX) [file pone.0187983.s003.docx]

The disjunct pattern of the Neotropical harvestman *Discocyrtus dilatatus* (Gonyleptidae) explained by climate-driven range shifts in the Quaternary: paleodistributional and molecular evidence

**S2 Table:** Relative importance of the 10 bioclimatic (bc) variables used to model the distribution of *Discocyrtus dilatatus*.

Variables are ordered according to the ‘sum score’ (percent contribution + permutation importance). The four top values for each estimator of relative importance are shaded, the highest one is underlined. Values correspond to the average, range and STD of a 30 replicates run made with MaxEnt.

| BC VARIABLES | percent contribution (avg) | min–max  ± STD | permutation importance (avg) | min–max  ± STD | SUM SCORE |
| --- | --- | --- | --- | --- | --- |
| bc4 - T° seasonality | **44.4049** | 40.7922 – 49.8960  ± 1.9313 | 16.0787 | 11.8150 – 25.7442  ± 2.7841 | 60.4836 |
| bc9 - mean T° driest quarter | 9.6986 | 5.2991 – 14.1965  ± 2.0215 | **36.4360** | 25.4136 – 45.1923  ± 5.2753 | 46.1346 |
| bc18 - precipitation warmest quarter | 26.3312 | 23.5963 – 28.6901  ± 1.4203 | 19.0569 | 9.1692 – 26.4957  ± 4.4655 | 45.3880 |
| bc3 - isothermality | 3.5036 | 1.1020 – 5.6578  ± 1.0056 | 16.4898 | 4.0238 – 33.1242  ± 7.3850 | 19.9933 |
| bc5 - max T° warmest month | 6.8969 | 5.9586 – 8.6741  ± 0.5538 | 4.1412 | 2.4290 – 6.8961  ± 1.0108 | 11.0380 |
| bc19 - precipitation coldest quarter | 3.8638 | 1.6376 – 6.9909  ± 1.1290 | 4.1897 | 1.7568 – 6.9720  ± 1.2901 | 8.0535 |
| bc15 - precipitation seasonality | 4.4046 | 1.9649 – 7.2008  ± 1.1750 | 2.0060 | 0.1680 – 5.0614  ± 1.4065 | 6.4106 |
| bc2 - mean monthly T° range | 0.1442 | 0.0018 – 0.4750  ± 0.1278 | 1.1078 | 0.0000 – 3.0599  ± 0.8593 | 1.2520 |
| bc13 - precipitation wettest month | 0.6982 | 0.0349 – 2.2144  ± 0.5500 | 0.1509 | 0.0000 – 0.9948  ± 0.2522 | 0.8491 |
| bc14 - precipitation driest month | 0.0541 | 0.0000 – 0.3164  ± 0.0722 | 0.3430 | 0.0000 – 1.0702  ± 0.3261 | 0.3972 |
