# Supplementary material for: The disjunct pattern of the Neotropical harvestman Discocyrtus dilatatus (Gonyleptidae) explained by climate-driven range shifts in the Quaternary: Paleodistributional and molecular evidence
Source: PLoS One. 2017 Nov 15;12(11):e0187983. doi: 10.1371/journal.pone.0187983 (PMC5687770; doi:10.1371/journal.pone.0187983)

The disjunct pattern of the Neotropical harvestman *Discocyrtus dilatatus* (Gonyleptidae) explained by climate-driven range shifts in the Quaternary: paleodistributional and molecular evidence

**S1 Fig: Map of population membership from a Bayesian population analysis in Geneland.**

The different colors indicate the four hypothetical populations inferred by the analyses.


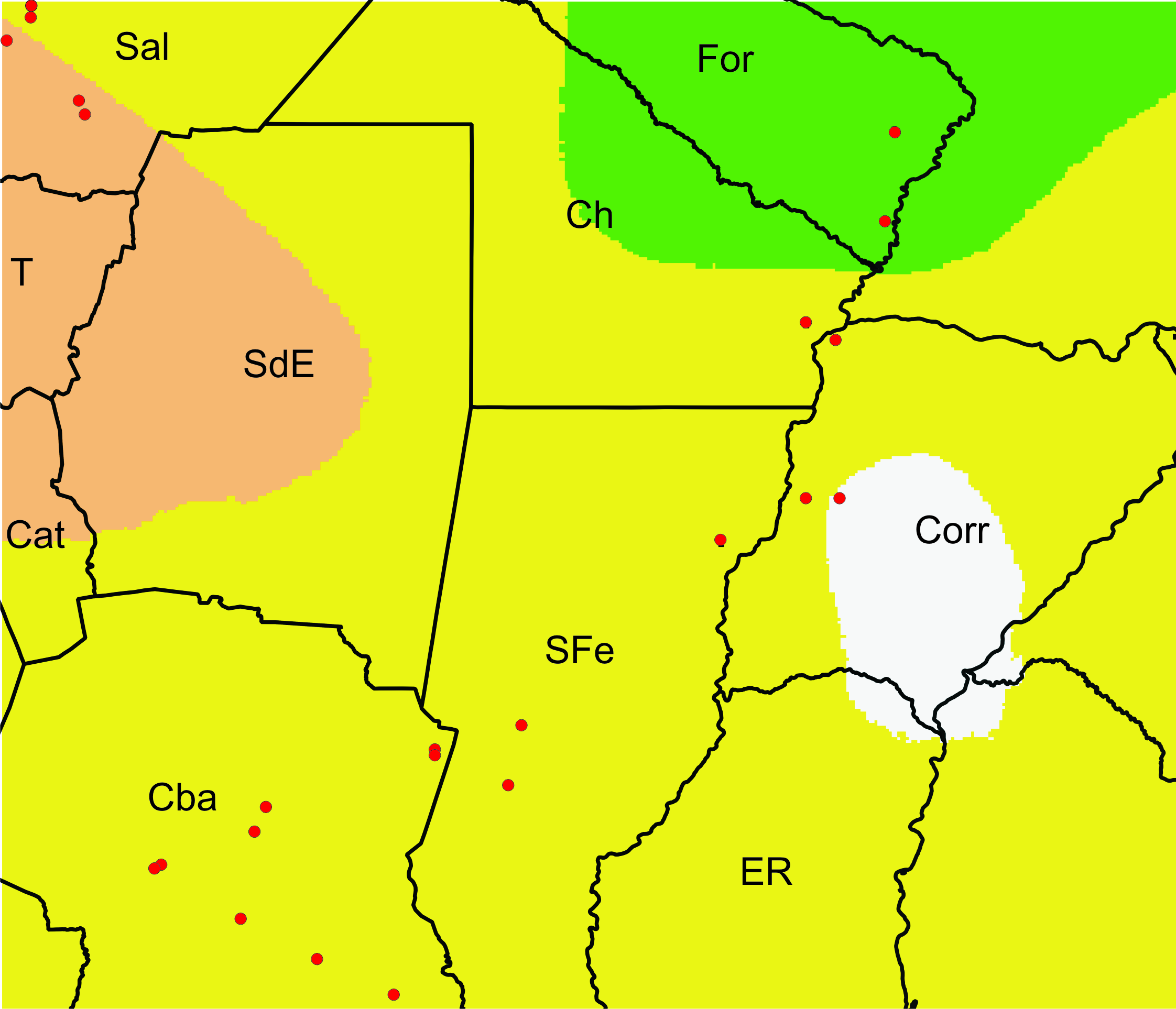

Supplement: S1 Fig — The different colors indicate the hypothetical populations (four in this case) inferred by the analyses. (DOCX) [file pone.0187983.s005.docx]
