# Supplementary material for: The disjunct pattern of the Neotropical harvestman Discocyrtus dilatatus (Gonyleptidae) explained by climate-driven range shifts in the Quaternary: Paleodistributional and molecular evidence
Source: PLoS One. 2017 Nov 15;12(11):e0187983. doi: 10.1371/journal.pone.0187983 (PMC5687770; doi:10.1371/journal.pone.0187983)

The disjunct pattern of the Neotropical harvestman *Discocyrtus dilatatus* (Gonyleptidae) explained by climate-driven range shifts in the Quaternary: paleodistributional and molecular evidence

**S2 Fig:** **Genealogical relationships of haplotypes of *Discocyrtus dilatatus.***

(A) Maximum likelihood (ML), (B) Maximum parsimony (MP), and (C) Bayesian Inference (BI). For each tree, nodes are given the corresponding statistical support value, if applicable (bootstrap over 50 for ML and MP, 1000 pseudoreplicates; posterior probability over 0.67 for BI). In (A) ML phylogeny, the support values for all three methods are given (ML bootstrap/MP bootstrap/BI posterior probability).


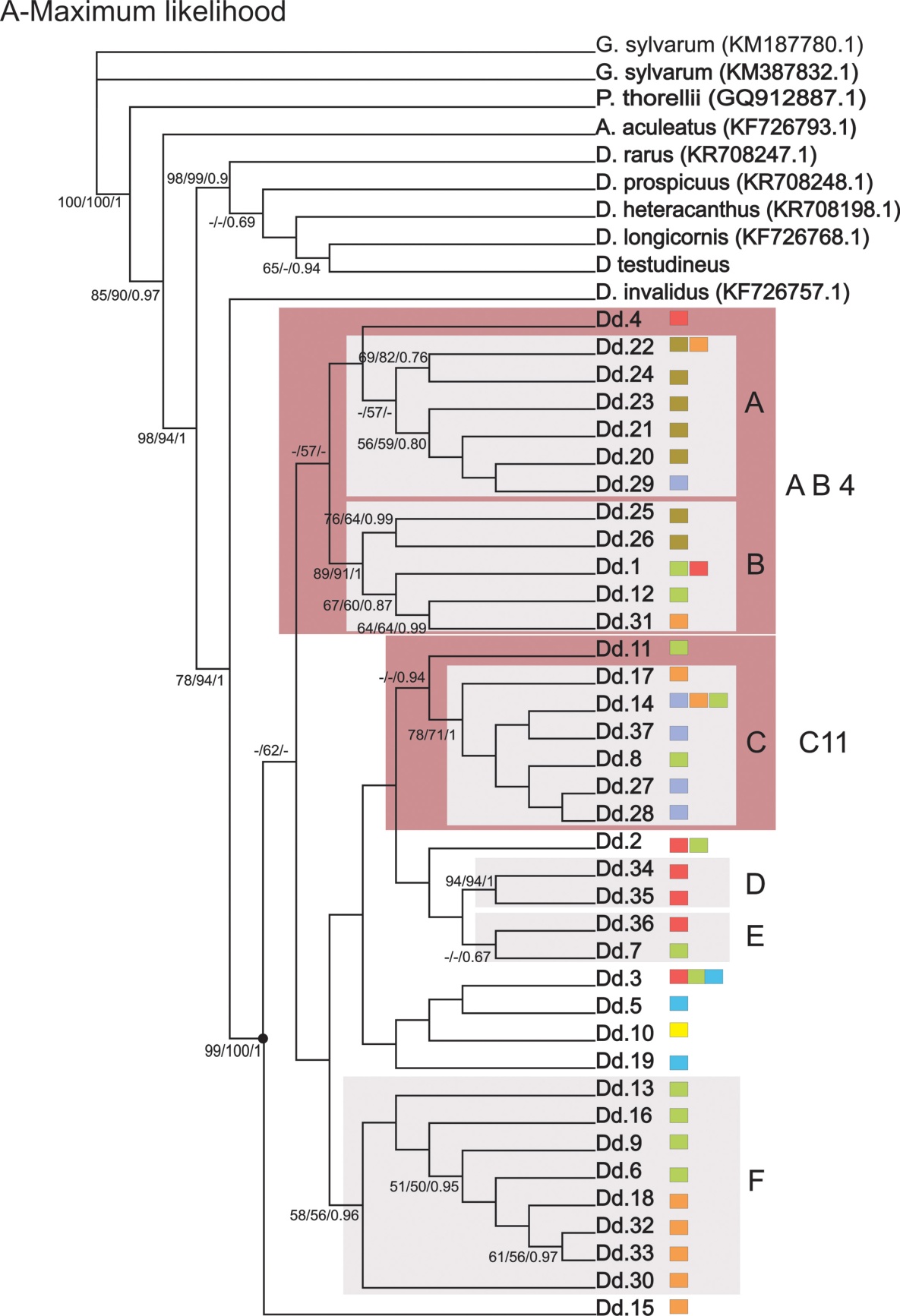


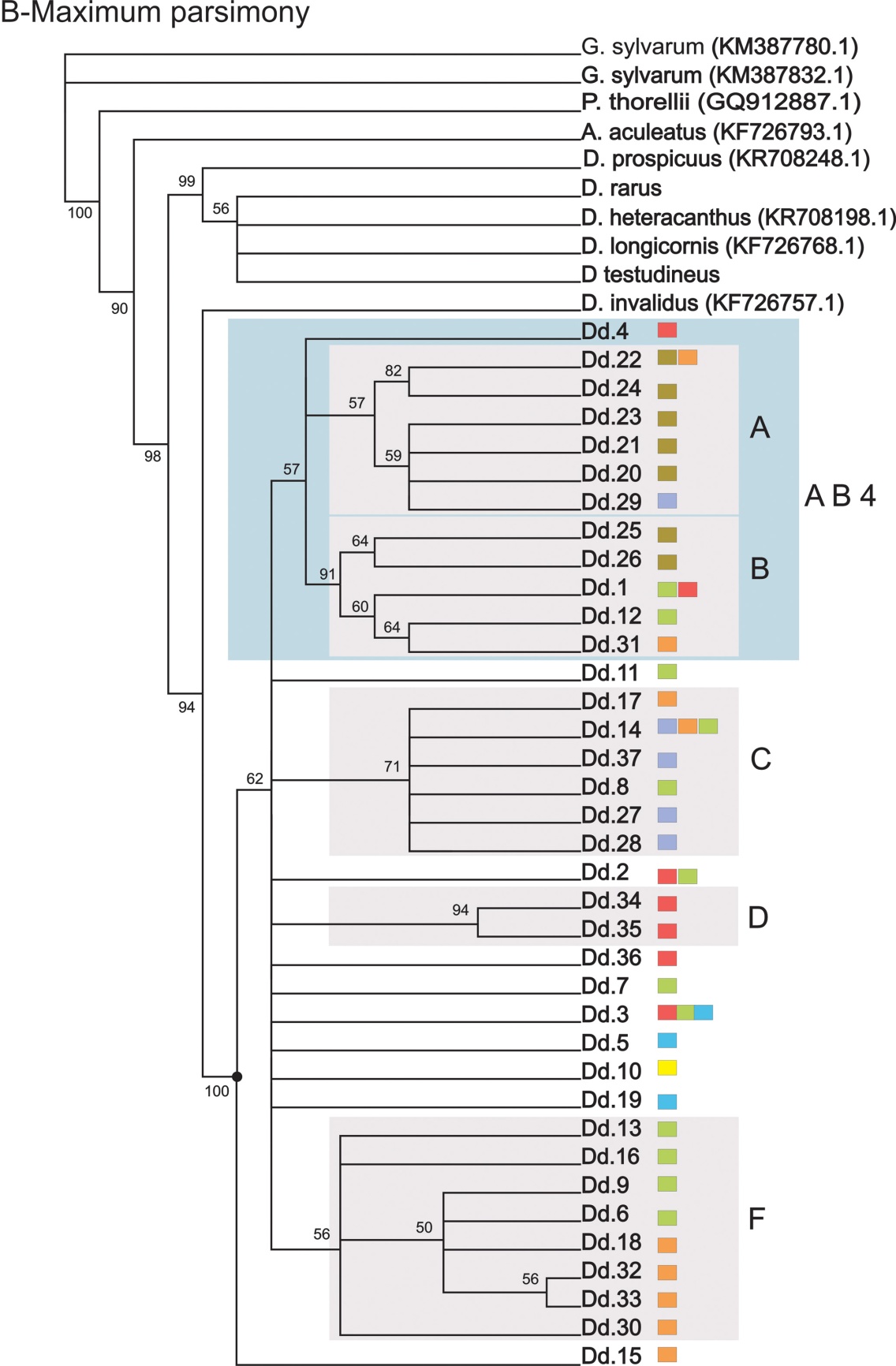


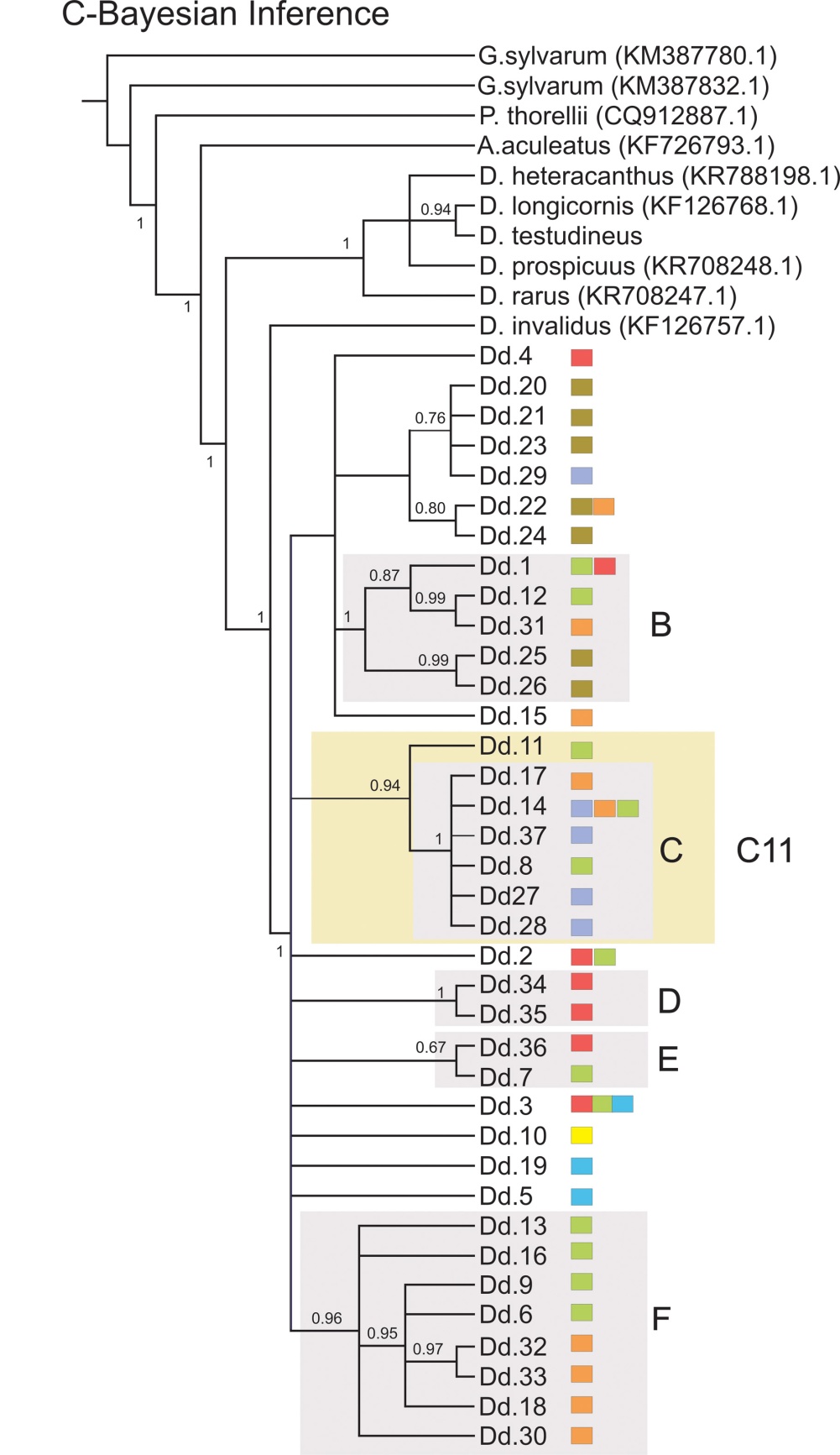

Supplement: S2 Fig — (A) Maximum likelihood (ML), (B) Maximum parsimony (MP), and (C) Bayesian Inference (BI). For each tree, nodes are given the corresponding statistical support value, if applicable (bootstrap over 50 for ML and MP, 1000 pseudoreplicates; posterior probability over 0.67 for BI). In (A) ML phylogeny, the support values for all three methods are given (ML bootstrap/MP bootstrap/BI posterior probability). (DOCX) [file pone.0187983.s006.docx]
